# Dramatic Transcriptional Changes in an Intracellular Parasite Enable Host Switching between Plant and Insect

**DOI:** 10.1371/journal.pone.0023242

**Published:** 2011-08-16

**Authors:** Kenro Oshima, Yoshiko Ishii, Shigeyuki Kakizawa, Kyoko Sugawara, Yutaro Neriya, Misako Himeno, Nami Minato, Chihiro Miura, Takuya Shiraishi, Yasuyuki Yamaji, Shigetou Namba

**Affiliations:** Department of Agricultural and Environmental Biology, Graduate School of Agricultural and Life Sciences, The University of Tokyo, Tokyo, Japan; University of Wisconsin-Milwaukee, United States of America

## Abstract

Phytoplasmas are bacterial plant pathogens that have devastating effects on the yields of crops and plants worldwide. They are intracellular parasites of both plants and insects, and are spread among plants by insects. How phytoplasmas can adapt to two diverse environments is of considerable interest; however, the mechanisms enabling the “host switching” between plant and insect hosts are poorly understood. Here, we report that phytoplasmas dramatically alter their gene expression in response to “host switching” between plant and insect. We performed a detailed characterization of the dramatic change that occurs in the gene expression profile of *Candidatus* Phytoplasma asteris OY-M strain (approximately 33% of the genes change) upon host switching between plant and insect. The phytoplasma may use transporters, secreted proteins, and metabolic enzymes in a host-specific manner. As phytoplasmas reside within the host cell, the proteins secreted from phytoplasmas are thought to play crucial roles in the interplay between phytoplasmas and host cells. Our microarray analysis revealed that the expression of the gene encoding the secreted protein PAM486 was highly upregulated in the plant host, which is also observed by immunohistochemical analysis, suggesting that this protein functions mainly when the phytoplasma grows in the plant host. Additionally, phytoplasma growth *in planta* was partially suppressed by an inhibitor of the MscL osmotic channel that is highly expressed in the plant host, suggesting that the osmotic channel might play an important role in survival in the plant host. These results also suggest that the elucidation of “host switching” mechanism may contribute to the development of novel pest controls.

## Introduction

Some pathogenic microorganisms can parasitise two quite different hosts. For example, malaria parasites can infect both human and mosquito vectors [1], and are rapidly transmitted by vectors over a wide area. Because this “host switching” is an essential stage in the life cycle of pathogens, it is important to determine its molecular mechanism(s) from the perspective of pest control.

Phytoplasmas (class *Mollicutes*, genus ‘*Candidatus* Phytoplasma’) are bacterial plant pathogens that have devastating effects on the yields of a wide range of low- and high-value crops and plants worldwide [2], [3]. Phytoplasmas infect more than 700 plant species and bring about marked changes in plant development, including witches' broom, dwarfism, and phyllody (Figure S1) [2], [4]. Despite their economic importance and biological features, phytoplasmas remain the most poorly characterised plant pathogens, primarily because efforts at *in vitro* culture, gene delivery, and mutagenesis have been unsuccessful [3].

The whole genome sequences were recently determined in four phytoplasma strains, i.e. ‘*Candidatus* Phytoplasma asteris’ (strain OY-M) [5], ‘*Candidatus* Phytoplasma asteris’ (strain AY-WB) [6] , ‘*Candidatus* Phytoplasma australiense’ (strain AUSGY) [7], and ‘*Candidatus* Phytoplasma mali’ (strain AT) [8]. Generally, phytoplasma has a small, reduced genome compared to other bacteria, e.g. OY-M phytoplasma genome is ca. 850 kbp in length, and encodes 756 genes. Like mycoplasmas [9], the phytoplasma genome lacks genes for amino acid biosynthesis, fatty acid biosynthesis, tricarboxylic acid cycle, and oxidative phosphorylation; however, the phytoplasma genome encodes even fewer metabolic function proteins than mycoplasmas. Especially, the phytoplasma has lost genes for the subunits of F_1_Fo type ATP synthase, which was previously believed to be necessary for cellular life. Phytoplasmas probably lost these biosynthesis genes as a result of reductive evolution adapted to a nutrient-rich environment as intracellular parasites [5].

The phytoplasma genome lacks homologues of the type III secretion system, which is essential for the virulence of many phytopathogenic bacteria [10]. Moreover, the phytoplasma possesses none of the known virulence genes found in other phytopathogenic bacteria. Because phytoplasmas lack most of the common metabolic pathways, it has been speculated that they must assimilate a wide range of materials from the host cells, probably with detrimental effects on the hosts. However, the molecular mechanism of phytoplasma disease remains unknown.

**Figure 1:**
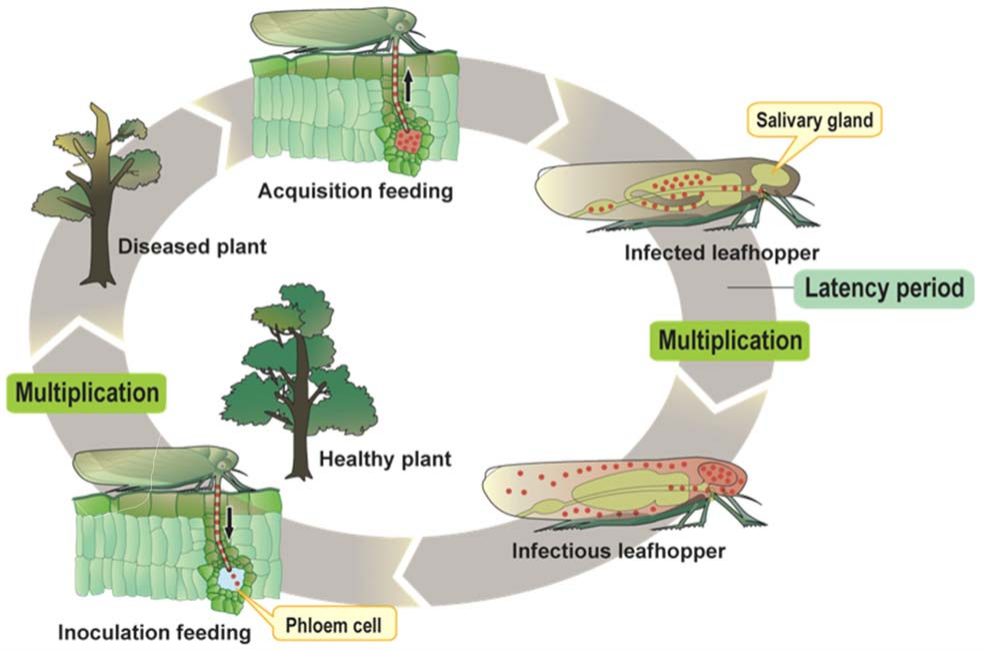
Life cycle of phytoplasmas. Phytoplasma is shown as a red dot. Phytoplasmas are unique biologically in that they can parasitise a diverse range of hosts, including plants (Kingdom Plantae) and insects (Kingdom Animalia). Phytoplasmas can reside endocellularly within the plant phloem and feeding insects (leafhoppers), and are spread among plants by insects.

Phytoplasmas are unique biologically in that they can parasitise a diverse range of hosts, including plants (Kingdom Plantae) and insects (Kingdom Animalia) [11] (Fig. 1). Phytoplasmas can reside endocellularly within the plant phloem and feeding insects (leafhoppers), and are spread among plants by insects. It is of interest how phytoplasmas can adapt to two diverse intracellular environments (*i.e.*, plant and insect cells). However, the mechanisms enabling the switch between plant and insect hosts are poorly understood.

In this study, we performed the first global gene expression analysis of phytoplasma. Our results indicate that phytoplasmas alter their gene expression in response to the plant and insect host. Moreover, we show that an inhibitor of a channel used in the plant host suppresses phytoplasma growth *in planta*.

## Results and Discussion

### Global gene expression profiling

**Figure 2:**
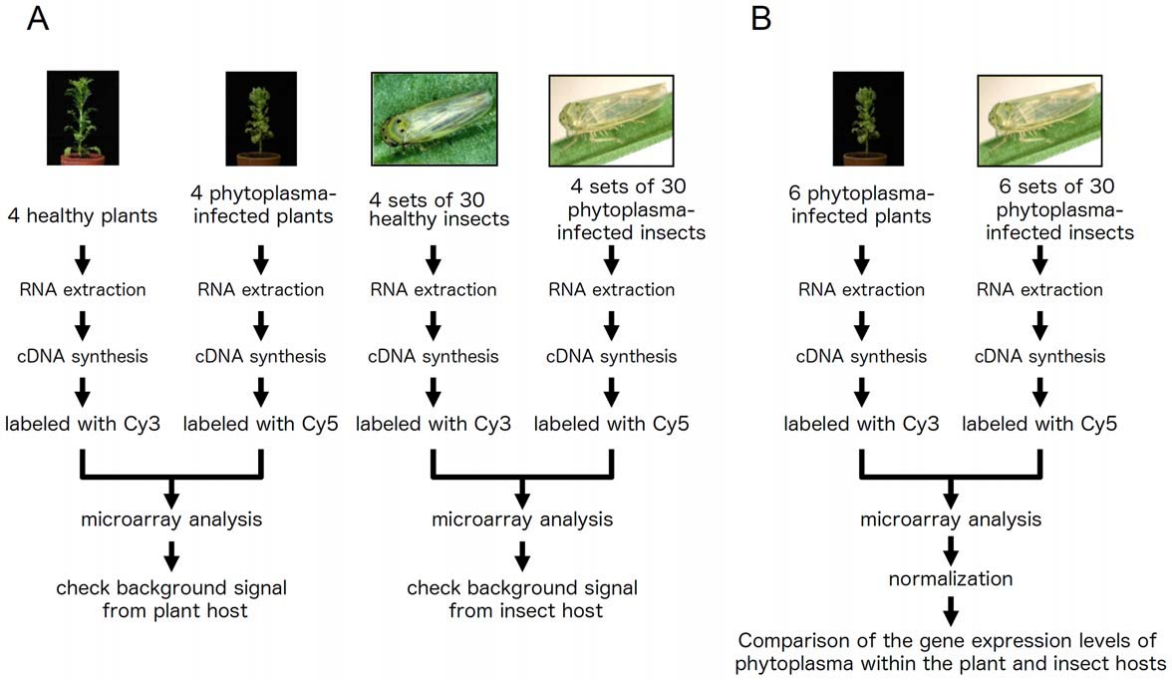
Experimental designs to compare the gene expressions between OY-M grown in plant and OY-M grown in insect host. (A) To evaluate the OY-M gene expression when grown in plant and to evaluate the background signal from plant host, total RNA was extracted from healthy plants and OY-M-infected plants, and labeled with Cy3 and Cy5, respectively. By subtracting the background signals of healthy plants, OY-M gene expression in plant host was obtained. Also, we obtained data on OY-M gene expression when grown in insect host by subtracting the background signals of healthy insect, and checked the background signal from insect host. (B) To investigate gene expression profiles between OY-M grown in plant host and OY-M grown in insect host, total RNA was extracted from OY-M-infected plants and OY-M-infected insects, and labelled with Cy3 and Cy5, respectively. These gene expression data were normalized by global normalization and compared.

To investigate gene expression levels for adaptation to diverse intracellular environments, the mRNA expression profiles of OY-M grown in a plant or insect host were evaluated using microarray analysis. As the population of phytoplasmas in a plant or insect host is quite small, we designed a highly sensitive phytoplasma microarray with 531 probes, each *ca*. 300 bp in length (the detailed information about the microarray design is shown in Table S1). First, since the populations of phytoplasmas in plant or insect hosts are quite small, we performed the preliminary microarray analysis. Total RNA was extracted from healthy plants and OY-M-infected plants, labelled with Cy3 and Cy5, respectively, and used for microarray analysis (Fig. 2A). We obtained the data on OY-M gene expression when grown in the plant host compared with the background signals of healthy plants. Likewise, we obtained data on OY-M gene expression when grown in the insect host by subtracting the background signals of healthy insects from those of OY-M-infected insects. As a result, although almost all probes were not hybridised with host's RNA, background signals from plant or insect host were detected in 13 probes (PAM035, PAM057, PAM080, PAM249, PAM250, PAM291, PAM300, PAM304, PAM419, PAM483, PAM575, PAM711 and PAM743) (Figure S2). Since the host's gene expression may be affected by the phytoplasma infection [12], [13], [14], there is a possibility that the background signals are different between the healthy host and the phytoplasma-infected host. However, we at least excluded these 13 genes from further analysis.

**Figure 3:**
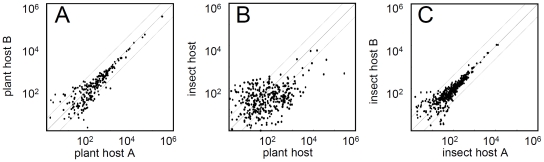
Comparison of signal intensities for phytoplasmas in plant and insect hosts. (A) Correlation between the gene expression levels of OY-M grown in plant hosts obtained from the experiment of Fig. 2A. The average intensities of two independent plants (plant host A) and the average intensities of rest two independent plants (plant host B) are plotted. (B) Correlation between the gene expression levels of OY-M grown in the plant and insect hosts. The average intensities of four independent experiments are plotted. (C) Correlation between the gene expression levels of OY-M grown in insect hosts. The average intensities of two independent insects (insect host A) and the average intensities of rest two independent insect (insect host B) are plotted. The dotted lines represent expression data within a 4-fold rate. Since almost all signals of “not infected” are zero (please see Figure S2), the plot of “infected” vs. “not infected” in the same host is not presented in this figure.

**Figure 4:**
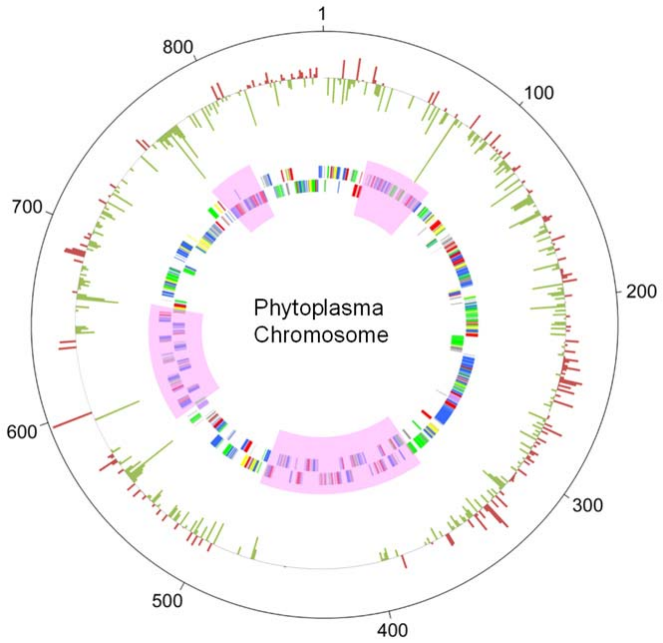
Circular representation of the OY-M phytoplasma chromosome and the ratio of expression level of each gene between in plant and in insect hosts. The outer circle shows the log2 ratio of expression level of each gene (expression level of OY-M grown in the plant host/expression level of OY-M grown in the insect host). Red bar shows high expression gene when grown in insect compared to when grown in plant. Green bar shows high expression gene when grown in plant compared to when grown in insect. The second circle shows predicted protein-coding regions on the plus strand. The third circle shows predicted protein-coding regions on the minus strand. Phage-like elements (called PMUs) are shown in pink.

Next, to investigate gene expression profiles between OY-M grown in a plant and OY-M grown in insect host, total RNA was extracted from OY-M-infected plants and OY-M-infected insects, labelled with Cy3 and Cy5, respectively, and used for microarray analysis (Fig. 2B). Six independent OY-M-infected plants and OY-M-infected insects were used in this study, and the expressional ratio of each gene between OY-M grown in plant and OY-M grown in insect was evaluated (Table S1). Surprisingly, 246 genes (ca. 33% of the genes in the genome) were differentially expressed between the two conditions (219 genes were up- or down-regulated more than two-fold between the two conditions (among them, 43 genes were statistically significant, *p*<0.05), 7 genes were detected only in OY-M grown in insect host, and 24 genes were detected only in OY-M grown in plant host). Of these 246 genes, 134 were upregulated in the plant host, whereas 112 were upregulated in the insect host (Table S1). This dramatic expressional change in host-switching was also supported by the comparison of signal intensities of the preliminary analysis (Fig. 3). Correlation coefficients of signal intensities among OY-M-infected plants were 0.993–0.999, and those among OY-M-infected insect were 0.919–0.982, while those between OY-M-infected plants and OY-M-infected insect were 0.115–0.158 (Fig. 3). It has been reported that several phage-like elements, called PMU (potential mobile unit), were found in the phytoplasma genome [6], [15]. However, PMUs include both genes expressed in plant host and those expressed in insect host, and bias against genomic region was not significantly recognized (Fig. 4).

**Figure 5:**
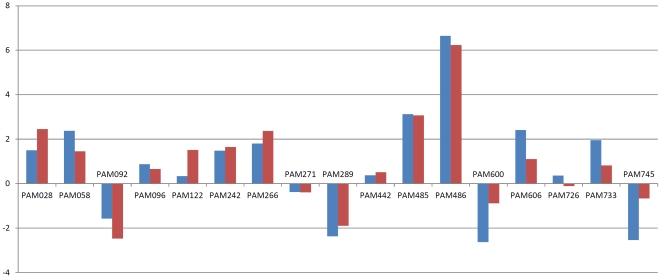
Comparison of gene expression between microarray (red) and real-time quantitative reverse transcription polymerase chain reaction (blue). Log ratio of signal in plant per signal in insect were indicated for PAM028 (*ibpA*; molecular chaperone), PAM058 (*mscL*; mechanosensitive channel), PAM242 (*himA*; bacterial nucleoid DNA-binding protein), PAM266 (hypothetical protein), PAM271 (hypothetical protein), PAM289 (membrane protein), PAM485 (hypothetical protein), PAM486 (secreted protein), PAM600 (*acoA*; thiamine pyrophosphate-dependent dehydrogenase), PAM606 (hypothetical protein), PAM726 (*ssb*; single-stranded DNA-binding protein) and PAM745 (*thrS*; threonyl-tRNA synthetase).

To examine the expression levels of the up- or downregulated genes suggested by the microarray experiments, we performed real-time quantitative reverse transcription polymerase reaction (qRT-PCR) for 17 selected genes. There was a high degree of concordance (*r* = 0.84) between the microarray data and the results of the qRT-PCR (Fig. 5; Figure S3). These results suggest that marked changes in gene expression occur in OY-M between plant and insect hosts.

### Transcription factors

**Figure 6:**
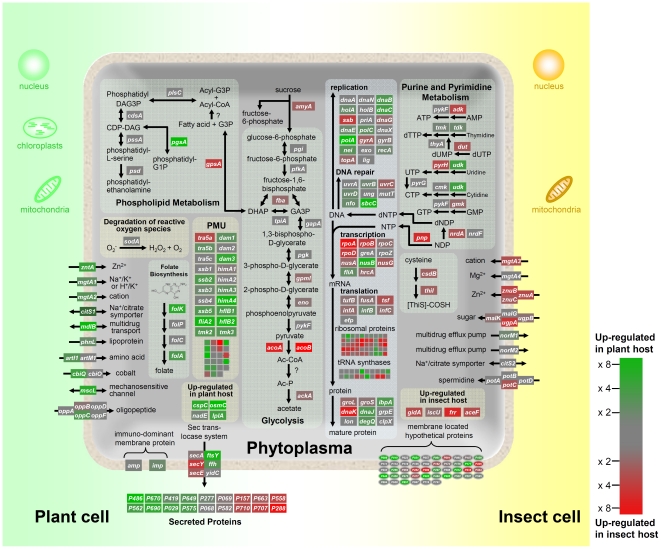
Overview of the phytoplasma metabolic pathways and the expression levels of each gene in plant and insect hosts. The genes upregulated in the plant host are shown in green, while those upregulated in the insect host are shown in red. Abbreviated gene names and their annotation list are shown in Table S1.

The sigma factor of RNA polymerase is involved in transcription initiation from specific promoter sequences. Most bacteria have multiple sigma factors that are required for complex cellular processes [16]. Although *Mollicutes* bacterial genomes generally encode few sigma factors [17], at least two sigma factors, *rpoD* and *fliA* (*rpoF*), have been identified in the OY-M genome [5]. Although it is possible that other hypothetical genes in the OY-M genome also encode transcription factors, these two sigma factors are thought to be involved in changing gene expression for adaptation to the environment. The *rpoD* gene of OY-M was upregulated, 4-fold, in the insect host (*p*<0.05). In contrast, the expression signal of the other sigma factor gene, *fliA*, was detected only in OY-M grown in the plant host (*p*<0.05) (Fig. 6, Table S1). These results suggest that *rpoD* activates the transcription of genes expressed specifically in the insect host, while *fliA* probably serves as a transcription factor for those expressed in the plant host.

### Transporters

The expression levels of several transporter genes were upregulated significantly depending on the plant or insect host (Fig. 6). For example, genes for the mechanosensitive channel, multidrug efflux pumps, and cobalt transporter were upregulated in the plant host, while the zinc, sugar, and oligopeptide transporters were upregulated in the insect host.

**Figure 7:**
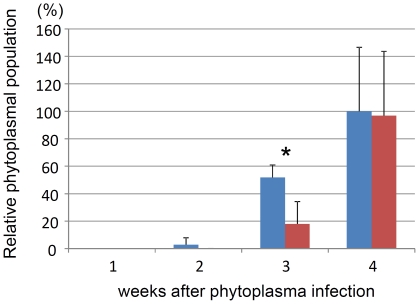
Growth suppression of phytoplasmas in planta on treatment with an inhibitor of the MscL osmotic channel. After treatment with 1 mM gadolinium chloride-containing water (red) or gadolinium chloride-free water (blue), the relative phytoplasma population was evaluated by real-time PCR using three independent leaves from each of three independent plants. The relative phytoplasma population of gadolinium chloride-free plants after 4 weeks are shown as 100%. Asterisk indicates that the phytoplasma populations were significantly different between gadolinium chloride-treated and not-treated samples (*p*<0.05, unpaired t-test).

The mechanisms by which cells can adjust to extremes of temperature, pH, and osmotic pressure are important for the survival of bacteria in the natural environment. The mechanosensitive MscL channel appears to sense mechanical stretching of the membrane and plays a fundamental role in protecting the cell from acute decreases in the osmolarity of the environment [18]. For example, In *Escherichia coli*, the expression level of the gene encoding the MscL channel is increased by twofold to threefold in media with high osmolarity [19]. In our microarray analysis, *mscL* gene expression was 5-fold higher when the phytoplasma infected a plant host compared with an insect host (*p*<0.05), suggesting that the MscL channel plays an important role in adaptation to the osmotic pressures of the plant-cell environment. To examine this hypothesis, we investigated a phytoplasma population in a plant host treated with an MscL channel inhibitor, gadolinium chloride [20]. Phytoplasma-inoculated plants were supplied with gadolinium chloride-free or gadolinium chloride-containing water, and samples of leaf tissues were collected from each plant at 1, 2, 3 and 4 weeks post inoculation. They were subjected to total DNA extraction and the real-time PCR assay to evaluate relative phytoplasma population. As a result, in the phytoplasma-infected plant supplied with gadolinium-free water, the phytoplasma population increases 3–30 fold per 1 week, which is consistent with the previous study [21]. In contrast, the growth of phytoplasma was suppressed by the inhibitor treatment at 3 weeks after phytoplasma infection (Fig. 7). At 4 weeks after phytoplasma infection, the phytoplasmal population was almost the same between the control plant and the inhibitor-containing water supplied plant (Fig. 7). These results suggest that the growth of the phytoplasma may be suppressed by the MscL channel inhibitor at early stage of phytoplasma infection, and imply that the MscL channel might play an important role in survival within a plant host cell. In general, photosynthetically synthesized carbon compounds, such as sugars and amino-nitrogen compounds, are the principal osmotic components of phloem sap, and are transported from the photosynthetic source leaves to heterotrophic sinks. Therefore, the osmotic pressure of phloem sap is quite different between photosynthetic source leaves and heterotrophic sinks [22]. Phytoplasma might express the *mscL* gene to adapt to these diverse osmotic pressures in the plant host.

The gene expressions for the zinc uptake transporters *znuABC* were detected only from OY-M in the insect host (Fig. 6). The total zinc concentration in eukaryotic cells ranges between 0.1 and 0.5 mM, although most of the zinc present inside eukaryotic cells is accumulated in vesicular sites or tightly bound to proteins [23]. Thus, little of the intracellular zinc within host cells is readily available to invading bacteria. In fact, intracellular growth was found to be decreased in *znuA*-deficient mutants of *Salmonella enterica* [24] and *Brucella abortus* [25]. Although the intracellular zinc concentration of the leafhopper is unknown, phytoplasmas may strongly express the genes for zinc-uptake transporters to adapt to an intracellular environment with a low zinc concentration.

One of the most intriguing discoveries in the genome analysis of phytoplasmas is the lack of genes encoding any of the subunits of the F_1_Fo type ATP synthase, which was previously believed to be necessary for cellular life [5], [3]. However, bioimaging analysis using potentiometric dyes suggests that the phytoplasma membrane has considerable potential [26], raising the question of how this potential is generated. There are five copies of P-type ATPase in the OY-M genome. One of these, *mgtA1*, is a P2C ATPase similar to the animal Na^+^/K^+^ and H^+^/K^+^ pumps, and was the first P2C ATPase identified in a prokaryote [27]. Thus, this ATPase may be involved in generating the membrane potential in phytoplasmas [27]. Interestingly, one of the five P-type ATPases (*mgtA3*) was upregulated in the insect host and two of P-type ATPases (*mgtA1* and *zntA*) were upregulated in the plant host (Fig. 6). These results suggest that phytoplasmas use these P-type ATPases for adaptation to the two different environments.

### Glycolysis

As phytoplasmas do not possess genes for F_1_Fo-ATP synthase, ATP biosynthesis is thought to be dependent on phosphorylation at the substrate level, such as in glycolysis [5], [28]. The expression levels of most glycolytic genes did not differ significantly in either the plant or insect host (Fig. 6), although three other glycolytic genes (*acoA*, *acoB* and *amyA*) were upregulated in the insect host. The *amyA* gene encodes sucrose phosphorylase, which catalyzes the conversion of sucrose to glucose-6-phosphate and fructose-6-phosphate. The *amyA* gene is encoded in the OY-M genome, but is truncated by a frameshift mutation, suggesting that this gene is not functional [5]. There is no *amyA* gene in the genome of *Ca.* Phytoplasma asteris AY-WB [6] or *Ca.* Phytoplasma mali [8]. In contrast, the *Ca.* Phytoplasma australiense genome possesses an intact, full-length *amyA* gene [7]. Thus, the ancestral phytoplasma may have had a functional *amyA* gene and have used sucrose, but some phytoplasma strains may have lost this gene in the course of evolution. Microarray analysis indicated that the *amyA* pseudogene was upregulated in the insect host (*p*<0.05). This indicates that the ancestor of OY-M might have used sucrose as a carbon source and expressed sucrose phosphorylase for its host adaptation, and that the expression of *amyA* gene might be still transcriptionally regulated even after it became a pseudogene.

### Antigenic membrane protein

The antigenic membrane protein Amp has been suggested to account for the major portion of the total cellular membrane protein fraction in most phytoplasmas [29]. Amp interacts with the microfilaments of its insect host *Macrosteles striifrons*, but not with those in leafhoppers that are unable to transmit OY, suggesting that the interaction between Amp and the insect microfilament complexes is involved in insect transmissibility [30]. These observations imply that Amp functions specifically in the insect host. However, the *amp* gene was neither upregulated nor downregulated in the plant and insect host. This suggests that amp gene may be not related to differential metabolism in the two hosts but probably related to strain specific binding to the host. Alternatively, in the plant host, Amp might be reserved for its function immediately after infection of an insect vector.

### Secreted proteins

**Figure 8:**
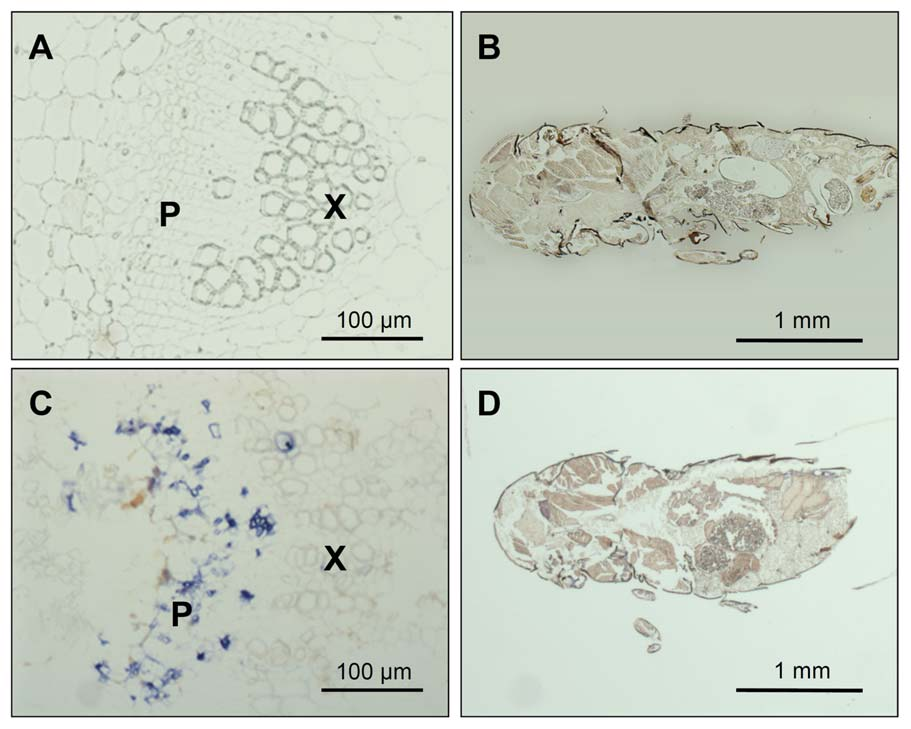
Localization of the phytoplasmal secreted protein, PAM486, detected by immunohistochemical analyses with anti-PAM486 antibody. X and P indicate xylem and phloem, respectively. (A) Immunohistochemical analyses of healthy garland chrysanthemum tissue section. (B) Immunohistochemical analyses of healthy leafhopper (*Macrosteles striifrons*) tissue section. (C) Immunohistochemical analyses of phytoplasma-infected garland chrysanthemum tissue section. (D) Immunohistochemical analyses of phytoplasma-infected leafhopper tissue section. Blue signals indicate the presence of the secreted protein PAM486. A phytoplasma-infected plant and a phytoplasma-infected insect were sectioned at 8 µm thickness.

As phytoplasmas reside within the host cell, the proteins secreted from phytoplasmas via the Sec translocation system function directly in the cytoplasm of the host cell [31]. Thus, these secreted proteins are thought to play crucial roles in the interplay between phytoplasmas and host cells [31], [32]. Our microarray analysis provides data on genes that are differentially regulated in plant and insect hosts. For example, the expression of the gene encoding the secreted protein PAM486 was upregulated 90-fold in the plant host (*p*<0.05) (Fig. 6; Table S1). To investigate the expression of PAM486 at the protein level, we observed the localisation of PAM486 by immunohistochemical analysis with an anti-PAM486 antibody. As a result, high expression of PAM486 protein was detected *in planta* (Fig. 8), however, in contrast, the expression of PAM486 protein was hardly detected in the insect host, which is in good agreement with the microarray data. From these results, we suggest that this protein functions mainly when the phytoplasma grows in the plant host. For example, we have recently reported that a secreted protein, TENGU, is highly expressed in the plant host, and induces phytoplasma-specific symptoms in plants, such as witches' broom and dwarfism [33]. Our microarray analysis provides important clues to the function of secreted proteins.

### Conclusions

Microarray analysis suggests that expression of the phytoplasma genome is regulated in response to the infectious stage in the plant or insect host. This is the first report on the global gene expression of intracellular bacteria in “host switching” between plant and insect hosts. The variation in phytoplasma gene expression in response to the diverse host environments is dramatic compared with those with environmental changes in other bacteria [34]. This may reflect the marked differences in the intracellular environments between plant and insect cells.

Further analyses are needed to determine the molecular mechanisms underlying the recognition of the two diverse environments. As the genes showing host-dependent expression likely play crucial roles in the infection of the respective hosts, our results may contribute to the development of novel methods of pest control for insect-transmissible pathogen diseases.

## Materials and Methods

### Phytoplasma-infected plants and insects

The mild symptom line of ‘*Candidatus* Phytoplasma asteris’ OY strain (OY-M) was maintained in garland chrysanthemum (*Chrysanthemum coronarium*), using the leafhopper vector *Macrosteles striifrons* [35]. Thirty-day-old leafhoppers carrying OY-M were used for the following experiments as OY-M-infected insect hosts. Healthy garland chrysanthemums were placed with leafhoppers carrying OY-M for 5–7 days. Then, the plants were sprayed to kill the insects and transferred to a greenhouse (20–30°C). About 1 month later, plants with distinct symptoms were used in the subsequent experiments as OY-M-infected plants.

### RNA isolation and quantitative real-time RT-PCR

Isogen reagent (Nippon Gene) was used to isolate total RNA from OY-M-infected plants and insects. To eliminate DNA contamination, the total RNA was treated with DNase I (Takara) before use in the microarray analysis or RT-PCR. Each of total RNA from five OY-M-infected insects (*M. striifrons*) and five plants (*C. coronarium*) was reverse-transcribed with a High-Capacity cDNA Reverse Transcription Kit (Applied Biosystems) according to the manufacturer's instructions. Quantitative real-time RT-PCR assays were performed using Thermal Cycler Dice® Real Time System (TaKaRa BIO INC.) and SYBR Green assay (SYBR® Premix Ex Taq™, TaKaRa BIO INC.). The tufB(F) primer (5′-GAA CAC ATT TTA TTA GCG CGC C-3′) and the tufB(R) primer (5′-TGT CGT CTC CTG GGA AAT CG-3′) were used for the quantification of the *tufB* gene expression as an internal standard [33]. Other primers used for the quantification of gene expressions are shown in Table S2.

To check that *tufB* is a suitable reference gene, we confirmed that *tufB* is stably expressed at the same level in both plant and insect host by estimating the expression levels of *tufB* by quantitative real-time RT-PCR using other genes (PAM472 (*rpsP*) and PAM163 (*ung*)) as an internal standard (Figure S4).

The threshold cycle (CT) value for each gene was normalized with the CT value of *tufB* gene using Multiplate RQ software (TaKaRa BIO INC.). Five OY-M-infected insects and five plants were used for the quantification of gene expression of each target gene. Standard curves of the *tufB* gene expression and the target gene expression were generated in each experiment. The relative value of the target gene expression against the *tufB* gene expression was obtained by three repetitions test, and the ratio of the target gene expression (the expression in plant host/the expression in insect host) was calculated.

### Construction of a microarray for phytoplasma

To investigate the gene expression levels in the phytoplasma, we designed and constructed a microarray. As the populations of phytoplasmas in plant or insect hosts are quite small, the concentration of phytoplasma RNA in the total RNA is usually less than 0.1%. Thus, probe length on the microarray is important for detecting phytoplasma gene expression. It has been reported that phytoplasma gene expression was detected by RNA blot hybridisation with *ca*. 300-bp probes [36]. Although the phytoplasma has 751 genes in its genome, we amplified 300-bp regions of 531 non-redundant genes, and constructed an IntelliGene chip microarray using these PCR products (TaKaRa BIO INC.). The detailed information about the microarray design, probe list and probe sequences is shown in Table S1.

### Preliminary microarray analysis

For the preliminary analysis, total RNA was extracted from four healthy and four OY-M-infected plants, and labelled with Cy3 and Cy5, respectively. Total RNA was also extracted from four groups of 30 healthy insects and four groups of 30 OY-M-infected insects, and labelled with Cy3 and Cy5, respectively. These four set of independent samples were prepared and hybridised with the microarrays by IntelliGene chip system (TaKaRa BIO INC.). 10 µg of total RNA with 100 pg lambda polyA RNA (internal control) was used for each microarray analysis. Cy3 or Cy5 labelled cDNA was synthesized using 300 pmol random primer by reverse transcriptase (TaKaRa BIO INC.). Hybridization was performed 14 hours at 65°C (6×SSC, 0.2% SDS, 5×Denhardt's solution). The hybridized slides were washed three times with 2×SSC/0.2% SDS (55°C), and washed one time with 0.05×SSC (at room temperature). The slides were scanned with an Affymetrix 428 Array Scanner (Affymetrix INC.). We used lambda internal control for normalisation and identified high background signal from host cell (more than 20 signal intensity). BioDiscovery ImaGene ver. 4.2 and the open source R software package (http://www.r-project.org) were used for processing and analysis of the microarray data. We also obtained the data on OY-M gene expression when grown in the plant host by subtracting the background signals of healthy plants from the signals of OY-M-infected plants, and obtained data on OY-M gene expression when grown in the insect host by subtracting the background signals of healthy insects from those of OY-M-infected insects (Fig. 3). Array data were deposited at the Gene Expression Omnibus (GEO) database of the National Center for Biotechnology (http://www.ncbi.nlm.nih.gov/geo) under the SubSeries GSE29274 (SuperSeries GSE30804).

### Microarray analysis

To investigate gene expression profiles between OY-M grown in a plant and OY-M grown in insect host, total RNA was extracted from six OY-M-infected plants and six groups of 30 OY-M-infected insects, and labelled with Cy3 and Cy5, respectively. These six set of independent samples were prepared and hybridised with the microarrays by IntelliGene chip system (TaKaRa BIO INC.). 10 µg of total RNA was used for each microarray analysis. The protocol of the hybridization is the same as the preliminary microarray analysis. The slides were scanned with an Affymetrix 428 Array Scanner (Affymetrix INC.). These gene expression data were normalised using the global normalisation method [37]. BioDiscovery ImaGene ver. 4.2 and the open source R software package (http://www.r-project.org) were used for processing and statistical analysis of the microarray data (one sample t-test) [38]. Array data were deposited at the GEO database of the National Center for Biotechnology under the SubSeries GSE30302 (SuperSeries GSE30804).

### Growth suppression of phytoplasmas in planta

Each of six healthy garland chrysanthemums was placed with eight leafhoppers carrying OY-M. Among them, three garland chrysanthemums were supplied with 1 mM gadolinium chloride (GdCl_3_) containing water [20], and three garland chrysanthemums were supplied with gadolinium chloride-free water. We obtained three leaves from each plant every week. Total DNA from each leaf was extracted with DNeasy Plant Mini Kit (QIAGEN) by the manufacturer's instructions. The relative phytoplasma population was evaluated by *tufB* gene-targeted real-time PCR as previously reported [39] with some modifications. The *tufB* gene-specific primer set Tuf1 (5′-GCTAA AACTT GTCCA CGTTG TACG-3′)/Tuf2 (5′-CGGAA ATAGA ATTGA GGACG GT-3′) were designed from partial sequences of the *tufB* gene of OY phytoplasma. Real-time quantitative PCR was performed using Thermal Cycler Dice® Real Time System (TaKaRa BIO INC.) and SYBR Green assay (SYBR® Premix Ex Taq™, TaKaRa BIO INC.). The reaction was carried out for up to 40 cycles using the following conditions: 5 sec at 95°C and 30 sec at 60°C. The results were analyzed using Multiplate RQ software (TaKaRa BIO INC.).

### Immunohistochemical analysis

To confirm the expression level of PAM486, immunohistochemical analyses were performed with anti-PAM486 antibody according to a described procedure [36]. Concentration of the anti- PAM486 antibodies used in plant tissue sections was 100 µg ml^−1^, and that used in insect tissue sections was 100 µg ml^−1^. Sections (∼2 cm in length) of stems were excised from healthy and OY-M-infected garland chrysanthemum. The stem pieces were fixed, embedded in paraffin, and cut into 8 µm sections with a microtome, PR-50 (Yamato Scientific, Saitama, Japan). Also, healthy and OY-M-infected insects were sectioned by the same methods. An Axio Imager Z1 microscope (Carl Zeiss MicroImaging GmbH, Jena, Germany) equipped with an AxioCam HRc camera (Carl Zeiss MicroImaging GmbH) controlled by AxioVision Rel. 4.6 software (Carl Zeiss MicroImaging GmbH) was used to collect images.

## Supporting Information

Figure S1 **Typical disease symtoms in a phytoplasma-infected hydrangea plant.** Left, a healthy hydrangea flower. Right, a phytoplasma-infected hydrangea flower showing phyllody (leaf-like petals and sepals).
(TIF)

Figure S2 **Heat map of signal intensities in microarray analysis.** Signal intensities from healthy plant, OY-M-infected plant, healthy insect and OY-M-infected insect were normalized with lambda polyA RNA (internal control), and were used for drawing the heat map. The gene IDs that signals were detected in healthy plant or healthy insect were indicated left.
(TIF)

Figure S3 **Correlation of log2 ratio of signal intensities (signal in plant host/signal in insect host) between microarray and real-time RT-PCR data.** 17 genes used in Fig. 5 were plotted. r, correlation coefficient.
(TIF)

Figure S4 **The expression levels of** ***tufB*** **gene in plant and insect host.** To check that *tufB* (PAM265) is a suitable reference gene, the expression levels of *tufB* in both plant (red) and insect host (blue) were estimated by quantitative real-time RT-PCR using other genes, (A) *rpsP* and (B) *ung*, as an internal standard. As a result, *tufB* is stably expressed at the same level in both plant and insect host. In contrast, PAM486 is highly expressed in plant host (p<0.01), which is consistent with the result when *tufB* is used as an internal standard.
(TIF)

Table S1 The microarray design and gene expression profiles of OY-M grown in between the plant and insect host.
(DOC)

Table S2 Primer sequence list used for qRT-PCR.
(DOC)
