# Supplementary figures and images for: Dramatic Transcriptional Changes in an Intracellular Parasite Enable Host Switching between Plant and Insect
Source: PLoS One. 2011 Aug 16;6(8):e23242. doi: 10.1371/journal.pone.0023242 (PMC3156718; doi:10.1371/journal.pone.0023242)

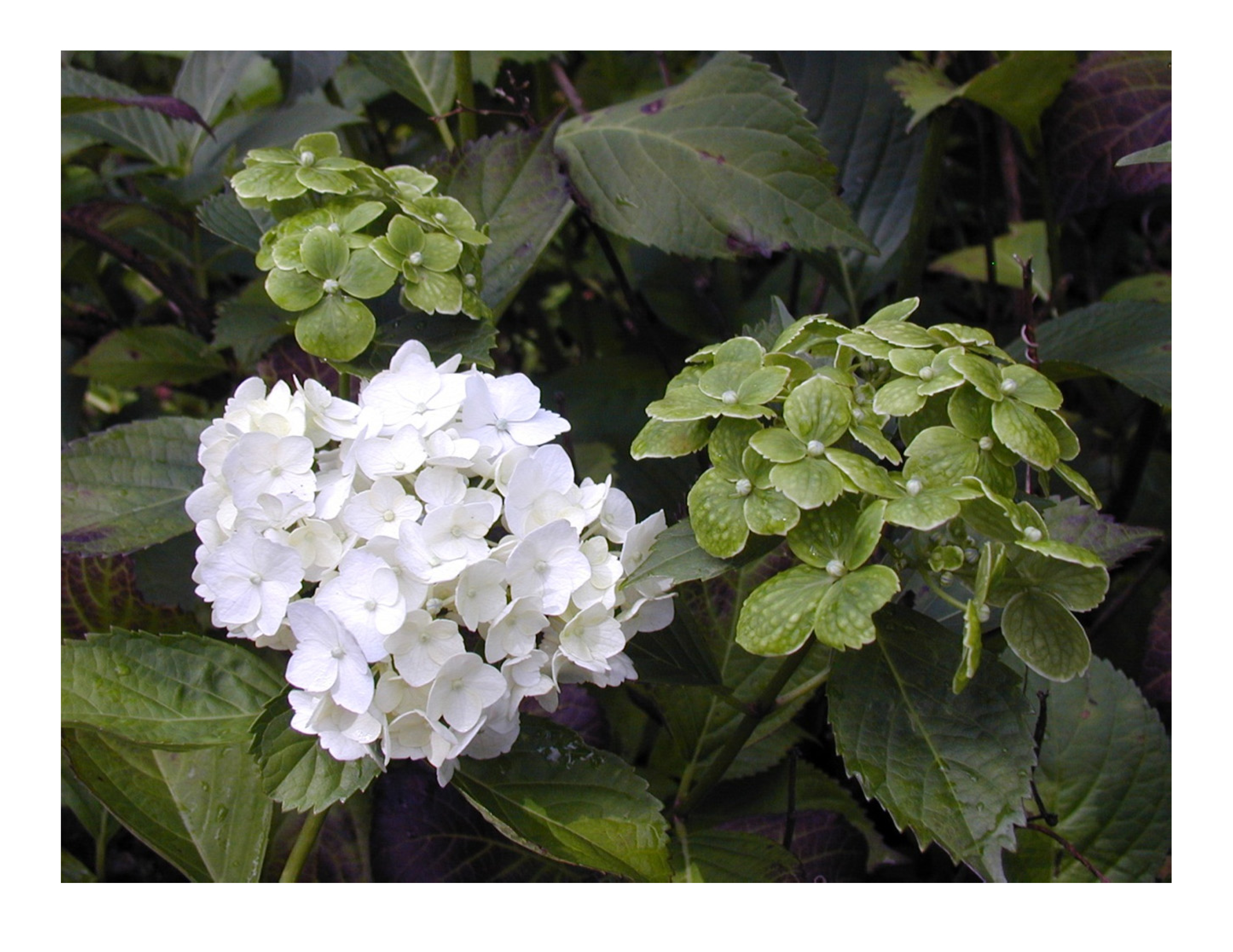

Supplement: Figure S1 — Typical disease symtoms in a phytoplasma-infected hydrangea plant. Left, a healthy hydrangea flower. Right, a phytoplasma-infected hydrangea flower showing phyllody (leaf-like petals and sepals). (TIF) [file pone.0023242.s001.tif]

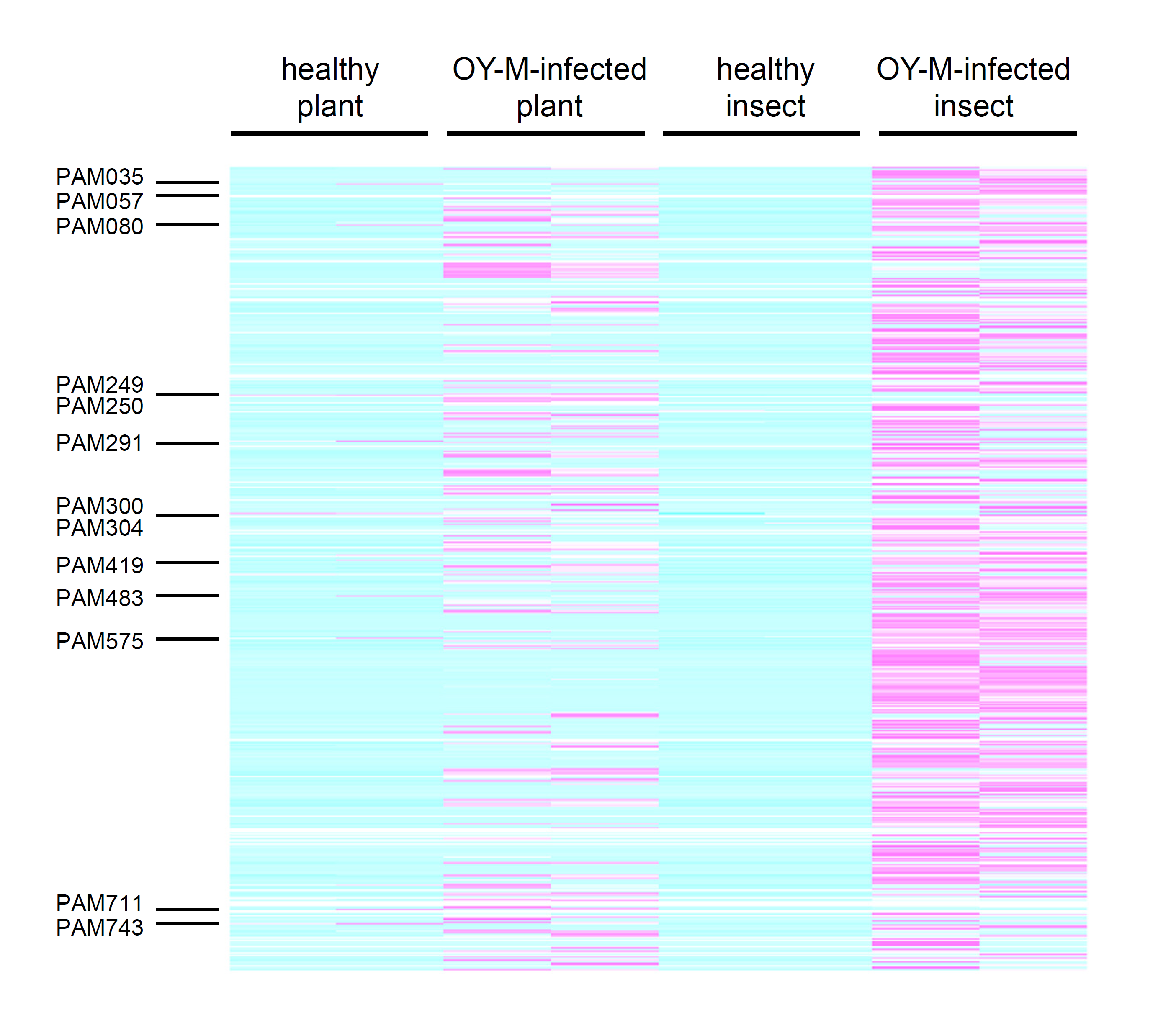

Supplement: Figure S2 — Heat map of signal intensities in microarray analysis. Signal intensities from healthy plant, OY-M-infected plant, healthy insect and OY-M-infected insect were normalized with lambda polyA RNA (internal control), and were used for drawing the heat map. The gene IDs that signals were detected in healthy plant or healthy insect were indicated left. (TIF) [file pone.0023242.s002.tif]

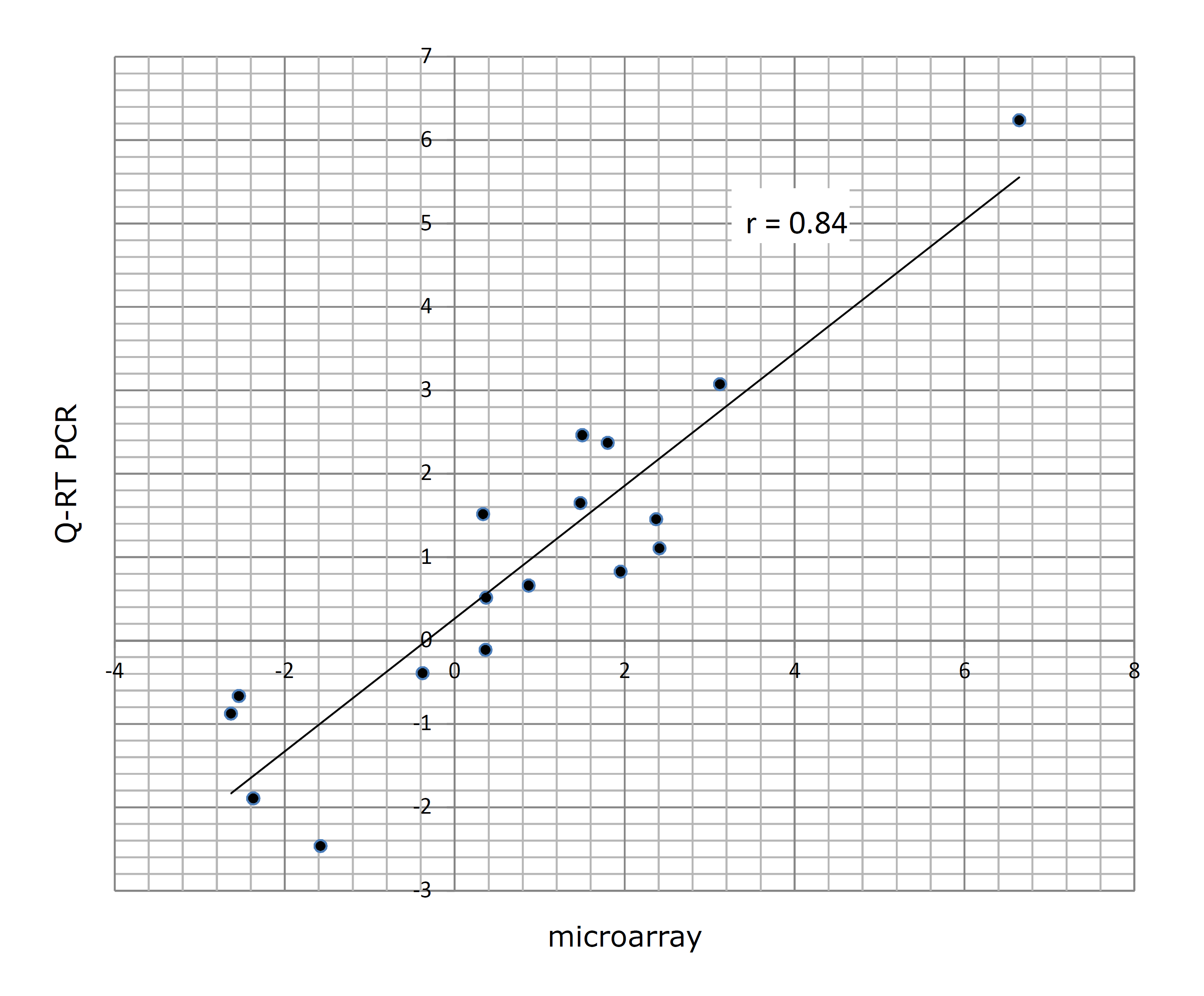

Supplement: Figure S3 — Correlation of log2 ratio of signal intensities (signal in plant host/signal in insect host) between microarray and real-time RT-PCR data. 17 genes used in Fig. 5 were plotted. r, correlation coefficient. (TIF) [file pone.0023242.s003.tif]

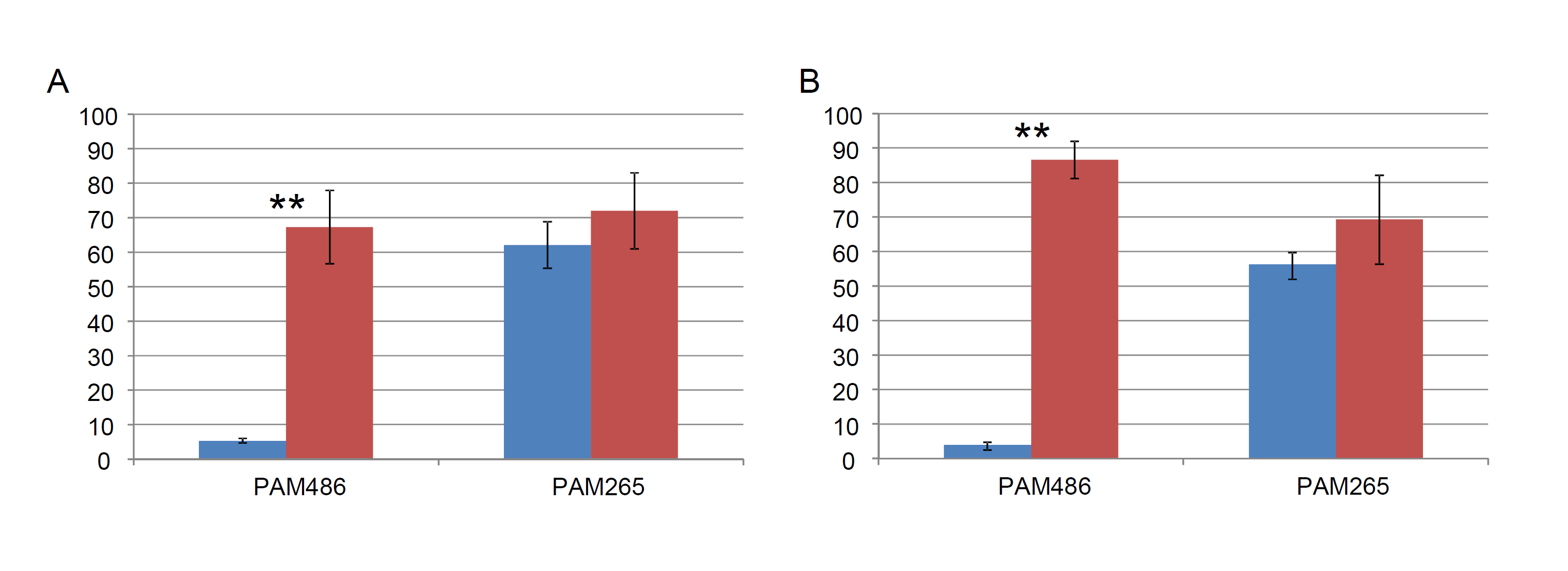

Supplement: Figure S4 — The expression levels of tufB gene in plant and insect host. To check that tufB (PAM265) is a suitable reference gene, the expression levels of tufB in both plant (red) and insect host (blue) were estimated by quantitative real-time RT-PCR using other genes, (A) rpsP and (B) ung, as an internal standard. As a result, tufB is stably expressed at the same level in both plant and insect host. In contrast, PAM486 is highly expressed in plant host (p<0.01), which is consistent with the result when tufB is used as an internal standard. (TIF) [file pone.0023242.s004.tif]
