# Supplementary material for: Dramatic Transcriptional Changes in an Intracellular Parasite Enable Host Switching between Plant and Insect
Source: PLoS One. 2011 Aug 16;6(8):e23242. doi: 10.1371/journal.pone.0023242 (PMC3156718; doi:10.1371/journal.pone.0023242)
Supplement: Table S1 — The microarray design and gene expression profiles of OY-M grown in between the plant and insect host. (DOC) [file pone.0023242.s005.doc]

Table S1

The microarray design and gene expression profiles of OY-M grown in between the plant and insect host. *P*-value was calculated by one sample t-test of six replicates. null, not detected. “plant” in Log_2_(signal in plant/signal in insect) column indicates a gene specifically expressed in plant host. “insect” in Log_2_(signal in plant/signal in insect) column indicates a gene specifically expressed in insect host.

| **PAM_no** | **Location_5'** | **Location_3'** | **Strand** | **gene index** | **Gene** | **Product** | **Amino acid length** | **Sequence length** | **Probe size** | **Log2 ratio of signal intensities (signal in plant/signal in insect)** | ***p*-value** |
| --- | --- | --- | --- | --- | --- | --- | --- | --- | --- | --- | --- |
| **PAM001** | 1 | 1524 | + | 39938486 | dnaA | chromosomal replication initiator protein | 507 | 1524 | 280 | null | - |
| **PAM002** | 1831 | 2967 | + | 39938486 | dnaN | DNA polymerase III beta subunit | 378 | 1137 | 305 | null | - |
| **PAM003** | 4447 | 4866 | + | 39938486 |  | hypothetical protein | 139 | 420 | 208 | -2.012 | 0.2144 |
| **PAM004** | 4948 | 5220 | + | 39938486 |  | hypothetical protein | 90 | 273 | 250 | null | - |
| **PAM005** | 5467 | 6222 | - | 39938486 | pgpB | membrane-associated phospholipid phosphatase | 251 | 756 | 213 | null | - |
| **PAM006** | 6431 | 7063 | - | 39938486 |  | hypothetical protein | 210 | 633 | 200 | -2.865 | - |
| **PAM007** | 7660 | 8976 | + | 39938486 | folC | folylpolyglutamate synthase | 438 | 1317 | 283 | null | - |
| **PAM008** | 10428 | 11675 | + | 39938486 | rfaG | 1,2-diacylglycerol 3-glucosyltransferase | 415 | 1248 | 282 | -0.270 | - |
| **PAM009** | 12171 | 12509 | + | 39938486 | rpsF | ribosomal protein S6 | 112 | 339 | 307 | -0.717 | 0.001856 |
| **PAM010** | 12524 | 12868 | + | 39938486 | ssb | single-stranded DNA-binding protein | 114 | 345 | 280 | 2.150 | 0.1667 |
| **PAM011** | 13006 | 13245 | + | 39938486 | rpsR | ribosomal protein S18 | 79 | 240 | 204 | -2.948 | 0.05333 |
| **PAM012** | 13316 | 15889 | + | 39938486 |  | exopolyphosphatase-related protein | 857 | 2574 | 283 | null | - |
| **PAM013** | 15886 | 17229 | + | 39938486 | dnaB | replicative DNA helicase | 447 | 1344 | 280 | -2.513 | 0.9942 |
| **PAM014** | 17300 | 18355 | + | 39938486 | prfA | protein chain release factor A | 351 | 1056 | 297 | -1.111 | 0.3989 |
| **PAM015** | 18448 | 19005 | + | 39938486 | sua5 | translation factor | 185 | 558 | 285 | -0.479 | 0.8714 |
| **PAM016** | 19044 | 20015 | - | 39938486 | holA | DNA polymerase III delta subunit | 323 | 972 | 298 | -0.811 | 0.4683 |
| **PAM017** | 20275 | 20865 | + | 39938486 |  | hypothetical protein | 196 | 591 | 313 | 2.579 | - |
| **PAM018** | 21671 | 23713 | + | 39938486 | valS | valyl-tRNA synthetase | 680 | 2043 | 304 | -0.255 | 0.7083 |
| **PAM019** | 23899 | 24366 | + | 39938486 |  | hypothetical protein | 155 | 468 | 291 | null | - |
| **PAM020** | 24424 | 26190 | + | 39938486 | cbiO | ABC-type cobalt transport system, ATPase component | 588 | 1767 | 294 | 0.154 | 0.5967 |
| **PAM022** | 28890 | 29273 | + | 39938486 | dppB | ABC-type dipeptide/oligopeptide transport system, permease component | 127 | 384 | 285 | -1.534 | - |
| **PAM023** | 29578 | 30684 | + | 39938486 |  | hypothetical protein | 368 | 1107 | 280 | null | - |
| **PAM024** | 30946 | 31737 | + | 39938486 |  | hypothetical protein | 263 | 792 | 288 | null | - |
| **PAM025** | 32258 | 33295 | + | 39938486 | dppD | ABC-type dipeptide/oligopeptide transport system, ATPase component | 345 | 1038 | 287 | 1.956 | - |
| **PAM026** | 33626 | 34087 | + | 39938486 | oppF | ABC-type dipeptide/oligopeptide transport system, ATPase component | 153 | 462 | 280 | 0.434 | 0.8435 |
| **PAM027** | 34179 | 37277 | - | 39938486 |  | hypothetical protein | 1032 | 3099 | 298 | -1.210 | 0.4902 |
| **PAM028** | 37635 | 38057 | + | 39938486 | ibpA | molecular chaperone | 140 | 423 | 312 | -1.312 | 0.02087 |
| **PAM029** | 38180 | 38635 | - | 39938486 |  | hypothetical protein | 151 | 456 | 296 | -1.382 | 0.0005 |
| **PAM030** | 38845 | 41607 | - | 39938486 | mgtA | cation transport ATPase | 920 | 2763 | 292 | -1.128 | 0.1344 |
| **PAM031** | 42625 | 43755 | + | 39938486 |  | hypothetical protein | 376 | 1131 | 288 | -2.939 | - |
| **PAM032** | 44868 | 45518 | + | 39938486 |  | hypothetical protein | 216 | 651 | 281 | null | - |
| **PAM033** | 45800 | 46258 | + | 39938486 |  | hypothetical protein | 152 | 459 | 211 | insect | - |
| **PAM035** | 47948 | 48301 | + | 39938486 |  | hypothetical protein | 117 | 354 | 309 | -2.814 | 0.004898 |
| **PAM037** | 48879 | 49766 | + | 39938486 | smc | chromosome segregation ATPase homolog | 295 | 888 | 283 | plant | - |
| **PAM038** | 50195 | 50506 | + | 39938486 |  | hypothetical protein | 103 | 312 | 293 | null | - |
| **PAM040** | 52707 | 53312 | + | 39938486 | fliA | DNA-directed RNA polymerase specialized sigma subunit | 201 | 606 | 298 | -1.147 | 0.01565 |
| **PAM046** | 58212 | 59057 | + | 39938486 |  | hypothetical protein | 281 | 846 | 280 | -1.569 | 0.06741 |
| **PAM050** | 61925 | 62146 | + | 39938486 |  | hypothetical protein | 73 | 222 | 207 | null | - |
| **PAM054** | 67342 | 69651 | + | 39938486 | nrdA | ribonucleotide reductase alpha subunit | 769 | 2310 | 300 | 1.340 | 0.161 |
| **PAM055** | 70037 | 71089 | + | 39938486 | nrdF | ribonucleotide reductase beta subunit | 350 | 1053 | 318 | -2.229 | 0.9704 |
| **PAM056** | 73506 | 73904 | + | 39938486 |  | hypothetical protein | 132 | 399 | 215 | 1.748 | - |
| **PAM057** | 74018 | 74953 | + | 39938486 |  | hypothetical protein | 311 | 936 | 308 | 0.008 | 0.9909 |
| **PAM058** | 75777 | 76295 | + | 39938486 | mscL | large-conductance mechanosensitive channel | 172 | 519 | 296 | -1.807 | 0.01549 |
| **PAM059** | 76501 | 78225 | + | 39938486 | mdlB | ABC-type multidrug/protein/lipid transport system, ATPase component | 574 | 1725 | 318 | null | 0.09089 |
| **PAM060** | 80717 | 81424 | + | 39938486 |  | hypothetical protein | 235 | 708 | 280 | -3.418 | - |
| **PAM061** | 81782 | 82084 | + | 39938486 |  | hypothetical protein | 100 | 303 | 250 | null | - |
| **PAM063** | 83594 | 83884 | + | 39938486 | himA | bacterial nucleoid DNA-binding protein | 96 | 291 | 257 | 0.422 | 0.8125 |
| **PAM064** | 84325 | 86229 | + | 39938486 | hflB | ATP-dependent Zn protease | 634 | 1905 | 282 | -1.592 | - |
| **PAM065** | 86315 | 86587 | + | 39938486 |  | hypothetical protein | 90 | 273 | 217 | -1.437 | 0.0998 |
| **PAM069** | 89402 | 90103 | + | 39938486 |  | hypothetical protein | 233 | 702 | 200 | 0.510 | - |
| **PAM070** | 90298 | 90870 | + | 39938486 |  | hypothetical protein | 190 | 573 | 309 | -8.818 | 0.1928 |
| **PAM071** | 91415 | 92062 | + | 39938486 |  | hypothetical protein | 215 | 648 | 292 | -0.695 | - |
| **PAM073** | 92749 | 93516 | + | 39938486 |  | hypothetical protein | 255 | 768 | 280 | -0.775 | 0.02286 |
| **PAM076** | 99183 | 100070 | + | 39938486 |  | hypothetical protein | 295 | 888 | 293 | 0.033 | - |
| **PAM077** | 100067 | 101188 | + | 39938486 | artM | ABC-type amino acid transport system, permease component | 373 | 1122 | 316 | null | - |
| **PAM078** | 101697 | 103502 | + | 39938486 |  | ABC-type amino acid transport system, permease component | 601 | 1806 | 282 | 1.943 | 0.9571 |
| **PAM079** | 103752 | 104231 | + | 39938486 | glnQ | ABC-type polar amino acid transport system, ATPase component | 159 | 480 | 307 | null | - |
| **PAM080** | 104409 | 104654 | + | 39938486 |  | hypothetical protein | 81 | 246 | 209 | -1.012 | 0.000838 |
| **PAM081** | 105231 | 105491 | + | 39938486 | rpsT | ribosomal protein S20 | 86 | 261 | 217 | -1.213 | 0.2673 |
| **PAM083** | 106430 | 107704 | + | 39938486 | serS | seryl-tRNA synthetase | 424 | 1275 | 287 | -0.868 | 0.9172 |
| **PAM084** | 107898 | 108221 | + | 39938486 |  | hypothetical protein | 107 | 324 | 299 | -1.566 | 0.1447 |
| **PAM085** | 108383 | 109348 | + | 39938486 |  | hypothetical protein | 321 | 966 | 281 | null | - |
| **PAM086** | 109503 | 109838 | + | 39938486 | rplU | ribosomal protein L21 | 111 | 336 | 296 | -0.522 | 0.686 |
| **PAM087** | 109844 | 110179 | + | 39938486 |  | hypothetical protein | 111 | 336 | 281 | 1.330 | 0.1982 |
| **PAM088** | 110181 | 110459 | + | 39938486 | rpmA | ribosomal protein L27 | 92 | 279 | 257 | -1.930 | 0.03485 |
| **PAM089** | 110486 | 111754 | + | 39938486 | obg | hypothetical protein | 422 | 1269 | 281 | -1.142 | 0.501 |
| **PAM090** | 112097 | 112297 | + | 39938486 | gepA | uncharacterized phage-associated protein | 66 | 201 | 283 | null | - |
| **PAM091** | 113366 | 114295 | - | 39938486 |  | hypothetical protein | 309 | 930 | 314 | 1.739 | 0.3707 |
| **PAM092** | 115631 | 116776 | + | 39938486 | znuA | ABC-type Mn/Zn transport system, periplasmic Mn/Zn-binding protein | 381 | 1146 | 285 | 1.574 | 0.1713 |
| **PAM093** | 116807 | 117571 | + | 39938486 | znuC | ABC-type Mn/Zn transport system, ATPase component | 254 | 765 | 280 | 1.201 | 0.1973 |
| **PAM094** | 117672 | 119831 | + | 39938486 | znuB | ABC-type Mn/Zn transport system, permease component | 719 | 2160 | 316 | 1.263 | 0.08479 |
| **PAM095** | 120521 | 121441 | + | 39938486 |  | hypothetical protein | 306 | 921 | 293 | 0.340 | 0.4393 |
| **PAM096** | 121578 | 123077 | + | 39938486 | phnL | ABC-type transport system, involved in lipoprotein release, ATPase component | 499 | 1500 | 293 | -1.130 | 0.001995 |
| **PAM097** | 123489 | 124316 | + | 39938486 | nlpA | ABC-type uncharacterized transport system, periplasmic component | 275 | 828 | 280 | 0.525 | 0.1513 |
| **PAM098** | 124440 | 124871 | + | 39938486 |  | hypothetical protein | 143 | 432 | 298 | -0.896 | 0.05914 |
| **PAM099** | 124840 | 125193 | + | 39938486 |  | hypothetical protein | 117 | 354 | 290 | null | - |
| **PAM100** | 125336 | 125773 | + | 39938486 | iscU | nifU-like protein | 145 | 438 | 284 | 1.140 | 0.3867 |
| **PAM101** | 125929 | 126663 | - | 39938486 |  | hypothetical protein | 244 | 735 | 281 | -0.872 | 0.2788 |
| **PAM102** | 127074 | 128312 | - | 39938486 | pcnB | tRNA nucleotidyltransferase/polyA polymerase | 412 | 1239 | 280 | -1.813 | 0.314 |
| **PAM103** | 128672 | 129157 | - | 39938486 |  | cytosine/adenosine deaminase | 161 | 486 | 299 | -0.411 | 0.388 |
| **PAM104** | 129216 | 129845 | - | 39938486 | tdk | thymidine kinase | 209 | 630 | 283 | -1.790 | 0.6079 |
| **PAM105** | 129965 | 130246 | - | 39938486 | rpmE | ribosomal protein L31 | 93 | 282 | 280 | -0.521 | 0.9986 |
| **PAM106** | 130471 | 130659 | - | 39938486 | rpmB | ribosomal protein L28 | 62 | 189 | 282 | -0.819 | 0.428 |
| **PAM107** | 131206 | 131559 | + | 39938486 |  | hypothetical protein | 117 | 354 | 292 | 0.444 | - |
| **PAM108** | 131721 | 132260 | + | 39938486 |  | hypothetical protein | 179 | 540 | 301 | 1.023 | - |
| **PAM109** | 133602 | 134309 | + | 39938486 |  | hypothetical protein | 235 | 708 | 297 | -2.797 | 0.4493 |
| **PAM110** | 134511 | 135191 | + | 39938486 |  | hypothetical protein | 226 | 681 | 280 | null | - |
| **PAM111** | 135268 | 135834 | + | 39938486 |  | hypothetical protein | 188 | 567 | 280 | null | - |
| **PAM112** | 135841 | 136182 | + | 39938486 |  | hypothetical protein | 113 | 342 | 280 | null | - |
| **PAM113** | 136537 | 136968 | + | 39938486 |  | hypothetical protein | 143 | 432 | 320 | -2.150 | - |
| **PAM114** | 137018 | 137326 | - | 39938486 |  | hypothetical protein | 102 | 309 | 232 | null | - |
| **PAM115** | 137373 | 138002 | + | 39938486 |  | hypothetical protein | 209 | 630 | 282 | 0.443 | - |
| **PAM116** | 138038 | 138400 | + | 39938486 |  | hypothetical protein | 120 | 363 | 280 | -4.490 | - |
| **PAM117** | 138877 | 140088 | + | 39938486 | hflB | ATP-dependent Zn protease | 403 | 1212 | 285 | null | - |
| **PAM118** | 140090 | 140833 | + | 39938486 |  | hypothetical protein | 247 | 744 | 283 | null | - |
| **PAM119** | 141035 | 141751 | + | 39938486 |  | hypothetical protein | 238 | 717 | 280 | 0.091 | 0.9354 |
| **PAM120** | 142019 | 142288 | + | 39938486 | groES | chaperonin GroES | 89 | 270 | 250 | 0.187 | 0.3184 |
| **PAM121** | 142480 | 144090 | + | 39938486 | groEL | chaperonin GroEL | 536 | 1611 | 295 | 0.535 | 0.4513 |
| **PAM122** | 144248 | 144949 | + | 39938486 | amp | antigenic membrane protein | 233 | 702 | 300 | -0.365 | 0.07083 |
| **PAM123** | 145355 | 146122 | + | 39938486 |  | hypothetical protein | 255 | 768 | 292 | -2.699 | 0.05243 |
| **PAM124** | 146315 | 147289 | + | 39938486 | nadE | NAD synthase | 324 | 975 | 303 | -2.414 | 0.6653 |
| **PAM125** | 147401 | 148537 | + | 39938486 | trmU | tRNA(5-methylaminomethyl-2-thiouridylate) methyltransferase | 378 | 1137 | 288 | -2.500 | 0.7973 |
| **PAM126** | 148452 | 150842 | + | 39938486 | spoT | guanosine polyphosphate pyrophosphohydrolase/synthetase | 796 | 2391 | 288 | -0.809 | 0.6027 |
| **PAM127** | 151153 | 152421 | + | 39938486 | hisS | histidyl-tRNA synthetase | 422 | 1269 | 315 | null | 0.7895 |
| **PAM128** | 152425 | 154185 | + | 39938486 | aspS | aspartyl-tRNA synthetase | 586 | 1761 | 318 | -0.339 | 0.2375 |
| **PAM129** | 154195 | 155505 | + | 39938486 | mesJ | hypothetical protein | 436 | 1311 | 281 | -0.028 | 0.2001 |
| **PAM131** | 157887 | 158798 | + | 39938486 | rluA | pseudouridylate synthases | 303 | 912 | 286 | -0.539 | 0.6954 |
| **PAM132** | 159000 | 159611 | + | 39938486 | abc | uncharacterized ABC-type transport system ATPase component | 203 | 612 | 307 | -3.931 | 0.3259 |
| **PAM133** | 159694 | 160737 | + | 39938486 | nlpA | ABC-type uncharacterized transport system, periplasmic component | 347 | 1044 | 280 | -2.645 | 0.4676 |
| **PAM134** | 160750 | 161451 | + | 39938486 |  | permease component of an uncharacterized ABC transporter | 233 | 702 | 280 | null | - |
| **PAM135** | 161563 | 161895 | + | 39938486 |  | hypothetical protein | 110 | 333 | 280 | 0.525 | 0.1091 |
| **PAM136** | 161971 | 162195 | + | 39938486 | rpsO | ribosomal protein S15P/S13E | 74 | 225 | 214 | 1.135 | 0.3234 |
| **PAM137** | 162349 | 162891 | + | 39938486 |  | hypothetical protein | 180 | 543 | 250 | 0.759 | 0.1391 |
| **PAM138** | 163245 | 163703 | + | 39938486 | rplM | ribosomal protein L13 | 152 | 459 | 284 | -0.171 | 0.2132 |
| **PAM139** | 163708 | 164100 | + | 39938486 | rpsI | ribosomal protein S9 | 130 | 393 | 319 | -0.765 | 0.02882 |
| **PAM140** | 164200 | 165549 | + | 39938486 | glnS | glutamyl- and glutaminyl-tRNA synthetase | 449 | 1350 | 294 | -0.251 | 0.4834 |
| **PAM141** | 165715 | 167031 | + | 39938486 | cysS | cysteinyl-tRNA synthetase | 438 | 1317 | 307 | -1.108 | 0.8973 |
| **PAM142** | 167203 | 168843 | + | 39938486 | rplM | ribosomal protein L13 | 546 | 1641 | 306 | -1.803 | - |
| **PAM143** | 169118 | 170422 | + | 39938486 | udk | uridine kinase | 434 | 1305 | 281 | 0.308 | 0.654 |
| **PAM144** | 170667 | 171371 | + | 39938486 | spoU | rRNA methylase | 234 | 705 | 290 | null | - |
| **PAM145** | 171465 | 172019 | + | 39938486 | ppa | inorganic pyrophosphatase | 184 | 555 | 306 | -1.643 | - |
| **PAM146** | 172267 | 172725 | + | 39938486 | smpB | tmRNA-binding protein | 152 | 459 | 283 | null | 0.2518 |
| **PAM147** | 172933 | 174789 | + | 39938486 |  | hypothetical protein | 618 | 1857 | 306 | 1.619 | 0.01303 |
| **PAM148** | 175032 | 176105 | + | 39938486 | nusA | transcription terminator | 357 | 1074 | 284 | -0.232 | 0.2694 |
| **PAM149** | 176111 | 176368 | + | 39938486 |  | hypothetical protein | 85 | 258 | 250 | 0.239 | 0.3088 |
| **PAM150** | 176528 | 178387 | + | 39938486 | infB | translation initiation factor 2 | 619 | 1860 | 282 | -1.822 | 0.4376 |
| **PAM151** | 178391 | 178732 | + | 39938486 | rbfA | ribosome-binding factor A | 113 | 342 | 306 | -0.778 | 0.001074 |
| **PAM152** | 179034 | 179600 | + | 39938486 | pth | peptidyl-tRNA hydrolase | 188 | 567 | 200 | 0.607 | - |
| **PAM153** | 179708 | 181000 | - | 39938486 |  | hemolysin | 430 | 1293 | 280 | -1.107 | 0.4925 |
| **PAM154** | 181476 | 183935 | - | 39938486 | priA | primosomal protein N' | 819 | 2460 | 280 | null | - |
| **PAM157** | 187236 | 188186 | - | 39938486 |  | hypothetical protein | 316 | 951 | 305 | 0.986 | 0.05757 |
| **PAM158** | 188497 | 189192 | + | 39938486 |  | hemolysin III homolog | 231 | 696 | 314 | -0.122 | 0.4682 |
| **PAM159** | 189434 | 190666 | + | 39938486 | ackA | acetate kinase | 410 | 1233 | 295 | -0.721 | 0.7031 |
| **PAM160** | 192132 | 192719 | + | 39938486 |  | hypothetical protein | 195 | 588 | 312 | -0.393 | 0.8265 |
| **PAM161** | 192871 | 193596 | - | 39938486 |  | hypothetical protein | 241 | 726 | 292 | -0.889 | 0.5563 |
| **PAM162** | 193875 | 194648 | + | 39938486 |  | hypothetical protein | 257 | 774 | 285 | 0.339 | 0.2902 |
| **PAM163** | 194730 | 195371 | + | 39938486 | ung | uracil DNA glycosylase | 213 | 642 | 288 | 0.334 | 0.4684 |
| **PAM164** | 195555 | 196007 | + | 39938486 | dut | dUTPase | 150 | 453 | 309 | 0.274 | 0.3271 |
| **PAM165** | 196610 | 197425 | + | 39938486 | rpsB | ribosomal protein S2 | 271 | 816 | 310 | 1.293 | 0.2086 |
| **PAM166** | 197515 | 198351 | + | 39938486 | tsf | translation elongation factor Ts | 278 | 837 | 316 | 0.655 | 0.349 |
| **PAM167** | 198441 | 199166 | + | 39938486 | pyrH | uridylate kinase | 241 | 726 | 292 | 0.030 | 0.4157 |
| **PAM168** | 199169 | 199723 | + | 39938486 | frr | ribosome recycling factor | 184 | 555 | 319 | 1.673 | 0.04388 |
| **PAM169** | 199795 | 200712 | + | 39938486 | cdsA | CDP-diglyceride synthetase | 305 | 918 | 292 | -3.546 | 0.9981 |
| **PAM170** | 200974 | 201798 | + | 39938486 | tpiA | triosephosphate isomerase | 274 | 825 | 301 | -0.054 | 0.9427 |
| **PAM171** | 201977 | 202774 | + | 39938486 | cof | hypothetical protein | 265 | 798 | 280 | -0.718 | 0.494 |
| **PAM172** | 203036 | 203902 | + | 39938486 | fba | fructose/tagatose bisphosphate aldolase | 288 | 867 | 293 | -0.328 | 0.3457 |
| **PAM173** | 204009 | 204341 | + | 39938486 |  | hypothetical protein | 110 | 333 | 312 | -1.943 | 0.7223 |
| **PAM174** | 204612 | 205808 | + | 39938486 | pgk | 3-phosphoglycerate kinase | 398 | 1197 | 320 | -1.521 | 0.682 |
| **PAM175** | 205969 | 206976 | + | 39938486 | gapA | glyceraldehyde-3-phosphate dehydrogenase | 335 | 1008 | 280 | 0.885 | 0.8347 |
| **PAM176** | 207358 | 208038 | + | 39938486 |  | hypothetical protein | 226 | 681 | 287 | -1.143 | 0.178 |
| **PAM177** | 208663 | 209136 | + | 39938486 |  | hypothetical protein | 157 | 474 | 320 | null | - |
| **PAM178** | 209279 | 210967 | + | 39938486 |  | oligoendopeptidase F | 562 | 1689 | 307 | 0.017 | 0.4471 |
| **PAM179** | 211167 | 211982 | + | 39938486 | cbiO | ABC-type cobalt transport system, ATPase component | 271 | 816 | 281 | -1.016 | 0.7622 |
| **PAM180** | 212828 | 213877 | + | 39938486 | cbiQ | ABC-type cobalt transport system, permease component | 349 | 1050 | 286 | -4.066 | 0.538 |
| **PAM181** | 213971 | 215221 | + | 39938486 | glyA | glycine hydroxymethyltransferase | 416 | 1251 | 320 | null | 0.4378 |
| **PAM182** | 215698 | 216618 | + | 39938486 |  | hypothetical protein | 306 | 921 | 298 | -1.217 | 0.05169 |
| **PAM183** | 216815 | 217906 | + | 39938486 |  | hypothetical protein | 363 | 1092 | 314 | null | - |
| **PAM184** | 218019 | 220721 | + | 39938486 | ileS | isoleucyl-tRNA synthetase | 900 | 2703 | 295 | -1.504 | 0.9554 |
| **PAM185** | 220738 | 221556 | + | 39938486 |  | uncharacterized BCR | 272 | 819 | 287 | 0.463 | 0.4824 |
| **PAM186** | 221711 | 224467 | + | 39938486 | mgtA | cation transport ATPase | 918 | 2757 | 310 | -0.276 | 0.4108 |
| **PAM187** | 224522 | 225679 | - | 39938486 |  | Zn-dependent carboxypeptidase | 385 | 1158 | 290 | null | - |
| **PAM188** | 225774 | 226019 | - | 39938486 |  | Zn-dependent carboxypeptidase | 81 | 246 | 202 | -1.306 | 0.5434 |
| **PAM189** | 226296 | 227099 | - | 39938486 | dppC | ABC-type dipeptide/oligopeptide system, permease component | 267 | 804 | 287 | -1.689 | 0.1192 |
| **PAM190** | 227128 | 228054 | - | 39938486 | dppB | ABC-type dipeptide/oligopeptide transport system, permease component | 308 | 927 | 286 | 0.574 | - |
| **PAM191** | 231479 | 233050 | - | 39938486 | oppA | ABC-type dipeptide/oligopeptide transport system, periplasmic component | 523 | 1572 | 316 | -1.745 | 0.966 |
| **PAM192** | 233256 | 234059 | - | 39938486 | dppD | ABC-type dipeptide/oligopeptide transport system, ATPase component | 267 | 804 | 305 | 0.466 | 0.8778 |
| **PAM193** | 234071 | 234652 | - | 39938486 |  | hypothetical protein | 193 | 582 | 280 | null | - |
| **PAM194** | 235406 | 238492 | - | 39938486 |  | hypothetical protein | 1028 | 3087 | 298 | -1.813 | 0.1837 |
| **PAM195** | 238797 | 239279 | - | 39938486 |  | hypothetical protein | 160 | 483 | 291 | null | 0.000618 |
| **PAM197** | 240541 | 240843 | + | 39938486 |  | hypothetical protein | 100 | 303 | 280 | 1.548 | 0.1231 |
| **PAM198** | 241162 | 241368 | + | 39938486 | rpmF | ribosomal protein L32 | 68 | 207 | 201 | -0.163 | 0.03055 |
| **PAM199** | 242717 | 243040 | + | 39938486 | rpsJ | ribosomal protein S10 | 107 | 324 | 280 | 0.284 | 0.09104 |
| **PAM200** | 243429 | 243902 | + | 39938486 | rplC | ribosomal protein L3 | 157 | 474 | 303 | -0.379 | 0.3123 |
| **PAM201** | 243889 | 244512 | + | 39938486 | rplD | ribosomal protein L4 | 207 | 624 | 313 | 0.087 | 0.2044 |
| **PAM202** | 244512 | 244802 | + | 39938486 | rplW | ribosomal protein L23 | 96 | 291 | 280 | 1.273 | 0.3035 |
| **PAM203** | 244862 | 245692 | + | 39938486 | rplB | ribosomal protein L2 | 276 | 831 | 288 | -0.262 | 0.2286 |
| **PAM204** | 245705 | 245986 | + | 39938486 | rpsS | ribosomal protein S19 | 93 | 282 | 253 | -0.708 | 0.2549 |
| **PAM205** | 246003 | 246392 | + | 39938486 | rplV | ribosomal protein L22 | 129 | 390 | 319 | 0.211 | 0.3802 |
| **PAM206** | 246376 | 247134 | + | 39938486 | rpsC | ribosomal protein S3 | 252 | 759 | 318 | 1.722 | 0.3526 |
| **PAM207** | 247112 | 247534 | + | 39938486 | rplP | ribosomal protein L16/L10E | 140 | 423 | 284 | 0.706 | 0.09637 |
| **PAM208** | 247531 | 247851 | + | 39938486 | rpmC | ribosomal protein L29 | 106 | 321 | 286 | 0.254 | 0.3235 |
| **PAM209** | 247851 | 248114 | + | 39938486 | rpsQ | ribosomal protein S17 | 87 | 264 | 250 | 0.620 | 0.2318 |
| **PAM210** | 248127 | 248489 | + | 39938486 | rplN | ribosomal protein L14 | 120 | 363 | 290 | 2.104 | 0.3066 |
| **PAM211** | 248611 | 248850 | + | 39938486 | rplX | ribosomal protein L24 | 79 | 240 | 218 | 0.569 | 0.3361 |
| **PAM212** | 248866 | 249411 | + | 39938486 | rplE | ribosomal protein L5 | 181 | 546 | 307 | 0.137 | 0.3805 |
| **PAM213** | 249426 | 249695 | + | 39938486 | rpsN | ribosomal protein S14 | 89 | 270 | 253 | 0.513 | 0.2337 |
| **PAM214** | 249717 | 250109 | + | 39938486 | rpsH | ribosomal protein S8 | 130 | 393 | 317 | -0.588 | 0.1352 |
| **PAM215** | 250156 | 250710 | + | 39938486 | rplF | ribosomal protein L6 | 184 | 555 | 306 | 0.465 | 0.2948 |
| **PAM216** | 250720 | 251073 | + | 39938486 | rplR | ribosomal protein L18 | 117 | 354 | 297 | 2.658 | 0.09844 |
| **PAM217** | 251088 | 251603 | + | 39938486 | rpsE | ribosomal protein S5 | 171 | 516 | 285 | 2.005 | 0.01674 |
| **PAM218** | 251600 | 251797 | + | 39938486 | rpmD | ribosomal protein L30/L7E | 65 | 198 | 299 | 0.116 | 0.1657 |
| **PAM219** | 251855 | 252235 | + | 39938486 | rplO | ribosomal protein L15 | 126 | 381 | 303 | 1.503 | 0.2402 |
| **PAM220** | 252238 | 253479 | + | 39938486 | secY | preprotein translocase subunit SecY | 413 | 1242 | 280 | 0.134 | 0.2354 |
| **PAM221** | 253498 | 254151 | + | 39938486 | adk | adenylate kinase | 217 | 654 | 301 | 2.064 | 0.1816 |
| **PAM222** | 254148 | 254891 | + | 39938486 | map | methionine aminopeptidase | 247 | 744 | 298 | -0.629 | 0.2946 |
| **PAM223** | 254967 | 255203 | + | 39938486 | infA | translation initiation factor IF-1 | 78 | 237 | 203 | -0.054 | 0.3378 |
| **PAM224** | 255216 | 255332 | + | 39938486 | rpmJ | ribosomal protein L36 | 38 | 117 | 200 | 0.613 | 0.2111 |
| **PAM225** | 255345 | 255710 | + | 39938486 | rpsM | ribosomal protein S13 | 121 | 366 | 282 | -0.173 | 0.8006 |
| **PAM226** | 255752 | 256138 | + | 39938486 | rpsK | ribosomal protein S11 | 128 | 387 | 318 | -0.435 | 0.4882 |
| **PAM227** | 256169 | 256912 | + | 39938486 | rpoA | DNA-directed RNA polymerase alpha subunit | 247 | 744 | 289 | 2.364 | 0.1144 |
| **PAM228** | 257206 | 257562 | + | 39938486 | rplQ | ribosomal protein L17 | 118 | 357 | 307 | -0.398 | 0.6524 |
| **PAM229** | 257734 | 258465 | + | 39938486 | truA | pseudouridylate synthase | 243 | 732 | 314 | -0.196 | 0.4747 |
| **PAM230** | 258513 | 259136 | + | 39938486 | tmk | thymidylate kinase | 207 | 624 | 309 | -0.965 | 0.2717 |
| **PAM231** | 259133 | 260122 | + | 39938486 | holB | DNA polymerase III, delta prime subunit | 329 | 990 | 284 | -0.080 | - |
| **PAM232** | 260206 | 261063 | + | 39938486 |  | hypothetical protein | 285 | 858 | 311 | -0.185 | 0.449 |
| **PAM233** | 261149 | 262711 | + | 39938486 | metG | methionyl-tRNA synthetase | 520 | 1563 | 288 | null | - |
| **PAM235** | 263801 | 264769 | + | 39938486 |  | hypothetical protein | 322 | 969 | 302 | 0.243 | 0.4626 |
| **PAM236** | 265227 | 265478 | + | 39938486 | trxA | thioredoxin | 83 | 252 | 200 | 0.214 | 0.9422 |
| **PAM237** | 265512 | 266363 | + | 39938486 | truB | pseudouridine synthase | 283 | 852 | 315 | -2.219 | 0.658 |
| **PAM238** | 266482 | 267219 | + | 39938486 | rsuA | 16S rRNA pseudouridylate synthase | 245 | 738 | 282 | -0.354 | 0.9653 |
| **PAM239** | 267259 | 267930 | + | 39938486 | cmk | cytidylate kinase | 223 | 672 | 291 | 0.955 | 0.7736 |
| **PAM240** | 267931 | 269367 | + | 39938486 |  | hypothetical protein | 478 | 1437 | 286 | -0.931 | 0.3644 |
| **PAM241** | 269418 | 270569 | + | 39938486 | gpsA | glycerol 3-phosphate dehydrogenase | 383 | 1152 | 317 | 2.381 | 0.03589 |
| **PAM242** | 270744 | 271028 | + | 39938486 | himA | bacterial nucleoid DNA-binding protein | 94 | 285 | 201 | -1.220 | 0.000346 |
| **PAM243** | 271525 | 272415 | + | 39938486 |  | hypothetical protein | 296 | 891 | 297 | 0.859 | 0.973 |
| **PAM244** | 272498 | 272647 | + | 39938486 | rpmG | ribosomal protein L33 | 49 | 150 | 250 | -0.694 | 4.61E-05 |
| **PAM245** | 272915 | 274171 | + | 39938486 | pepP | xaa-Pro aminopeptidase | 418 | 1257 | 283 | -0.183 | 0.909 |
| **PAM246** | 274923 | 276347 | + | 39938486 | proS | prolyl-tRNA synthetase | 474 | 1425 | 290 | -0.766 | 0.5166 |
| **PAM247** | 276574 | 276708 | + | 39938486 | rpmH | ribosomal protein L34 | 44 | 135 | 289 | 1.153 | 0.3062 |
| **PAM248** | 276812 | 277237 | + | 39938486 | rnpA | ribonuclease P | 141 | 426 | 295 | -2.163 | 0.4679 |
| **PAM249** | 277256 | 278833 | + | 39938486 | yidC | preprotein translocase subunit YidC | 525 | 1578 | 280 | 0.644 | 0.4693 |
| **PAM250** | 282933 | 283574 | - | 39938486 |  | hypothetical protein | 213 | 642 | 280 | -0.114 | 0.04592 |
| **PAM251** | 284635 | 286782 | + | 39938486 | pnp | polyribonucleotide nucleotidyltransferase | 715 | 2148 | 290 | 0.377 | 0.2069 |
| **PAM252** | 286852 | 290022 | + | 39938486 | mgtA | cation transport ATPase | 1056 | 3171 | 307 | 1.261 | 0.1847 |
| **PAM253** | 290245 | 290430 | + | 39938486 | rpmG | ribosomal protein L33 | 61 | 186 | 319 | -1.468 | 0.1386 |
| **PAM254** | 290447 | 290857 | + | 39938486 | secE | preprotein translocase subunit SecE | 136 | 411 | 281 | 1.059 | 0.009216 |
| **PAM255** | 290859 | 291473 | + | 39938486 | nusG | transcription antiterminator | 204 | 615 | 299 | 0.626 | 0.1804 |
| **PAM256** | 291671 | 292096 | + | 39938486 | rplK | ribosomal protein L11 | 141 | 426 | 286 | -1.185 | 0.7038 |
| **PAM257** | 292157 | 292882 | + | 39938486 | rplA | ribosomal protein L1 | 241 | 726 | 319 | 0.460 | 0.1799 |
| **PAM258** | 292922 | 293431 | + | 39938486 | rplJ | ribosomal protein L10 | 169 | 510 | 292 | 1.068 | 0.1055 |
| **PAM259** | 293443 | 293853 | + | 39938486 | rplL | ribosomal protein L7/L12 | 136 | 411 | 298 | -0.368 | 0.6294 |
| **PAM260** | 294025 | 297846 | + | 39938486 | rpoB | DNA-directed RNA polymerase beta subunit | 1273 | 3822 | 306 | 0.829 | 0.05702 |
| **PAM261** | 297849 | 301910 | + | 39938486 | rpoC | DNA-directed RNA polymerase beta' subunit | 1353 | 4062 | 286 | -0.126 | 0.532 |
| **PAM262** | 301931 | 302350 | + | 39938486 | rpsL | ribosomal protein S12 | 139 | 420 | 316 | -0.541 | 0.5804 |
| **PAM263** | 302439 | 302909 | + | 39938486 | rpsG | ribosomal protein S7 | 156 | 471 | 280 | -0.097 | 0.4524 |
| **PAM264** | 302934 | 305000 | + | 39938486 | fusA | translation elongation factor EF-G | 688 | 2067 | 304 | -0.253 | 0.5808 |
| **PAM265** | 305131 | 306315 | + | 39938486 | tufB | translation elongation factor EF-Tu | 394 | 1185 | 320 | 0.130 | 0.2878 |
| **PAM266** | 306887 | 307555 | + | 39938486 |  | hypothetical protein | 222 | 669 | 292 | -0.866 | 0.01058 |
| **PAM267** | 307603 | 308241 | - | 39938486 | gidB | S-adenosylmethionine-dependent methyltransferase | 212 | 639 | 281 | 0.149 | 0.1205 |
| **PAM268** | 308228 | 310081 | - | 39938486 | gidA | glucose inhibited division protein A | 617 | 1854 | 298 | 0.177 | 0.16 |
| **PAM269** | 310138 | 310833 | - | 39938486 |  | hypothetical protein | 231 | 696 | 286 | 0.261 | 0.1666 |
| **PAM270** | 311826 | 312911 | + | 39938486 |  | uncharacterized BCR | 361 | 1086 | 303 | 0.325 | 0.1961 |
| **PAM271** | 313230 | 313541 | + | 39938486 |  | hypothetical protein | 103 | 312 | 293 | 0.490 | 0.4381 |
| **PAM272** | 314047 | 315633 | + | 39938486 |  | hypothetical protein | 528 | 1587 | 318 | 0.153 | 0.301 |
| **PAM273** | 315658 | 316119 | + | 39938486 |  | uncharacterized BCR | 153 | 462 | 285 | -1.060 | 0.906 |
| **PAM274** | 316094 | 316279 | + | 39938486 |  | hypothetical protein | 61 | 186 | 234 | -0.084 | 0.259 |
| **PAM275** | 316651 | 317526 | + | 39938486 | miaA | tRNA delta(2)-isopentenylpyrophosphate transferase | 291 | 876 | 303 | -1.524 | 0.9268 |
| **PAM276** | 317585 | 318154 | + | 39938486 | efp | translation elongation factor EF-P | 189 | 570 | 311 | -2.249 | 0.9406 |
| **PAM277** | 318371 | 319135 | + | 39938486 |  | hypothetical protein | 254 | 765 | 311 | -3.030 | 0.8488 |
| **PAM278** | 319165 | 320052 | + | 39938486 |  | hypothetical protein | 295 | 888 | 317 | -0.341 | 0.9758 |
| **PAM279** | 320143 | 322086 | + | 39938486 | topA | topoisomerase IA | 647 | 1944 | 286 | 0.674 | 0.5389 |
| **PAM280** | 322195 | 323790 | - | 39938486 | norM | Na+-driven multidrug efflux pump | 531 | 1596 | 280 | -0.053 | 0.4719 |
| **PAM281** | 324839 | 325837 | + | 39938486 | pfkA | 6-phosphofructokinase | 332 | 999 | 305 | -0.902 | 0.7462 |
| **PAM282** | 325982 | 327466 | + | 39938486 |  | hypothetical protein | 494 | 1485 | 313 | 1.874 | - |
| **PAM283** | 327589 | 328869 | + | 39938486 | pgi | glucose-6-phosphate isomerase | 426 | 1281 | 320 | -1.135 | 0.8056 |
| **PAM284** | 329038 | 330330 | + | 39938486 | eno | enolase | 430 | 1293 | 281 | -0.154 | 0.6998 |
| **PAM285** | 330452 | 331990 | + | 39938486 | gpmI | phosphoglyceromutase | 512 | 1539 | 316 | 0.336 | 0.4373 |
| **PAM286** | 332415 | 333560 | + | 39938486 | citS | malate/citrate symporter | 381 | 1146 | 291 | -0.019 | 0.8011 |
| **PAM287** | 333629 | 334969 | + | 39938486 | pykF | pyruvate kinase | 446 | 1341 | 281 | -1.248 | 0.8986 |
| **PAM288** | 335094 | 336308 | - | 39938486 |  | hypothetical protein | 404 | 1215 | 281 | 3.622 | 0.01296 |
| **PAM289** | 336401 | 337345 | - | 39938486 |  | hypothetical protein | 314 | 945 | 285 | 2.709 | 0.000698 |
| **PAM290** | 337963 | 339339 | + | 39938486 | argE | acetylornithine deacetylase | 458 | 1377 | 280 | 0.723 | 0.7052 |
| **PAM291** | 339373 | 339753 | + | 39938486 |  | hypothetical protein | 126 | 381 | 307 | -1.378 | 0.01385 |
| **PAM292** | 339884 | 340327 | + | 39938486 | argE | acetylornithine deacetylase | 147 | 444 | 293 | 0.049 | 0.8526 |
| **PAM293** | 340372 | 341238 | + | 39938486 | thyA | thymidylate synthase | 288 | 867 | 280 | 1.078 | 0.4805 |
| **PAM294** | 341235 | 341726 | + | 39938486 | folA | dihydrofolate reductase | 163 | 492 | 313 | -2.107 | 0.02875 |
| **PAM295** | 341720 | 342529 | + | 39938486 | plsC | 1-acyl-sn-glycerol-3-phosphate acyltransferase | 269 | 810 | 282 | 0.363 | 0.7829 |
| **PAM296** | 342630 | 343058 | + | 39938486 |  | hypothetical protein | 142 | 429 | 286 | 2.244 | 0.2541 |
| **PAM297** | 343147 | 343494 | - | 39938486 |  | hypothetical protein | 115 | 348 | 265 | -0.934 | 0.000404 |
| **PAM298** | 343499 | 343714 | - | 39938486 |  | uncharacterized BCR | 71 | 216 | 201 | -1.647 | 0.03716 |
| **PAM299** | 343768 | 344328 | - | 39938486 |  | N6-adenine-specific methylase | 186 | 561 | 289 | -0.789 | 0.5068 |
| **PAM300** | 344325 | 344924 | - | 39938486 |  | hypothetical protein | 199 | 600 | 288 | 1.099 | 0.05158 |
| **PAM301** | 344911 | 345480 | - | 39938486 |  | hypothetical protein | 189 | 570 | 271 | -0.575 | 0.3724 |
| **PAM302** | 345470 | 345982 | - | 39938486 | greA | transcription elongation factor | 170 | 513 | 284 | -1.013 | 0.9448 |
| **PAM303** | 345993 | 346577 | - | 39938486 | smtA | SAM-dependent methyltransferase | 194 | 585 | 300 | null | - |
| **PAM304** | 346579 | 347034 | - | 39938486 |  | Hollyday junction resolvase | 151 | 456 | 308 | -0.118 | 0.5146 |
| **PAM305** | 347381 | 349975 | - | 39938486 | alaS | alanyl-tRNA synthetase | 864 | 2595 | 290 | -0.781 | - |
| **PAM306** | 350438 | 351805 | + | 39938486 | ksgA | dimethyladenosine transferase | 455 | 1368 | 284 | -1.570 | 0.3527 |
| **PAM308** | 353275 | 353958 | - | 39938486 |  | hypothetical protein | 227 | 684 | 316 | -0.378 | 0.6195 |
| **PAM309** | 354084 | 355100 | - | 39938486 | lplA | lipoate-protein ligase A | 338 | 1017 | 284 | -1.145 | 0.05725 |
| **PAM310** | 356125 | 356589 | + | 39938486 |  | hypothetical protein | 154 | 465 | 281 | null | - |
| **PAM312** | 358034 | 358504 | + | 39938486 |  | hypothetical protein | 156 | 471 | 200 | 1.422 | 0.1756 |
| **PAM313** | 358467 | 359141 | + | 39938486 |  | hypothetical protein | 224 | 675 | 280 | 1.511 | 0.152 |
| **PAM315** | 360616 | 363732 | + | 39938486 | uvrD | ATP-dependent DNA helicase | 1038 | 3117 | 280 | -0.717 | 0.2863 |
| **PAM318** | 366112 | 366591 | - | 39938486 |  | hypothetical protein | 159 | 480 | 280 | -1.512 | 0.1782 |
| **PAM321** | 368215 | 368709 | + | 39938486 |  | hypothetical protein | 164 | 495 | 291 | 0.233 | 0.1526 |
| **PAM322** | 368724 | 369350 | + | 39938486 |  | hypothetical protein | 208 | 627 | 286 | -0.712 | 0.5675 |
| **PAM324** | 370112 | 370441 | + | 39938486 | ssb | single-stranded DNA-binding protein | 109 | 330 | 304 | -0.379 | 0.8333 |
| **PAM332** | 377580 | 377939 | + | 39938486 |  | hypothetical protein | 119 | 360 | 280 | null | - |
| **PAM337** | 383271 | 384200 | - | 39938486 | tra5 | putative transposase | 309 | 930 | 312 | 1.610 | - |
| **PAM339** | 384989 | 385417 | - | 39938486 |  | hypothetical protein | 142 | 429 | 289 | -0.814 | 0.3679 |
| **PAM342** | 388385 | 390145 | - | 39938486 |  | retron-type reverse transcriptase | 586 | 1761 | 287 | null | - |
| **PAM344** | 391588 | 392202 | - | 39938486 | tmk | thymidylate kinase | 204 | 615 | 306 | -1.358 | 0.004924 |
| **PAM345** | 392276 | 392905 | - | 39938486 |  | hypothetical protein | 209 | 630 | 320 | -0.869 | 0.08804 |
| **PAM346** | 393437 | 394282 | - | 39938486 |  | hypothetical protein | 281 | 846 | 300 | -0.250 | 0.8027 |
| **PAM347** | 394311 | 395036 | - | 39938486 |  | hypothetical protein | 241 | 726 | 280 | -1.069 | 0.06898 |
| **PAM382** | 428227 | 428550 | + | 39938486 |  | hypothetical protein | 107 | 324 | 257 | insect | - |
| **PAM395** | 440956 | 441615 | - | 39938486 | dam | site-specific DNA methylase | 219 | 660 | 307 | 0.128 | 0.6794 |
| **PAM400** | 444994 | 445443 | + | 39938486 |  | hypothetical protein | 149 | 450 | 287 | null | - |
| **PAM411** | 457519 | 458022 | - | 39938486 |  | hypothetical protein | 167 | 504 | 310 | -1.175 | 0.08129 |
| **PAM412** | 458035 | 458352 | - | 39938486 |  | hypothetical protein | 105 | 318 | 250 | -2.617 | 0.4552 |
| **PAM418** | 465284 | 466435 | - | 39938486 |  | hypothetical protein | 383 | 1152 | 299 | null | - |
| **PAM419** | 466441 | 467253 | - | 39938486 |  | hypothetical protein | 270 | 813 | 280 | -0.934 | 0.08183 |
| **PAM420** | 468176 | 468508 | - | 39938486 | himA | bacterial nucleoid DNA-binding protein | 110 | 333 | 280 | null | 0.8556 |
| **PAM428** | 474466 | 475764 | + | 39938486 | dnaB | replicative DNA helicase | 432 | 1299 | 282 | -0.005 | 0.9907 |
| **PAM432** | 478646 | 480664 | - | 39938486 | uvrB | helicase subunit of the DNA excision repair complex | 672 | 2019 | 288 | -2.685 | 0.662 |
| **PAM433** | 481032 | 482417 | - | 39938486 | uvrC | nuclease subunit of the excinuclease complex | 461 | 1386 | 296 | 1.023 | - |
| **PAM434** | 482970 | 483836 | + | 39938486 | pdxK | pyridoxal/pyridoxine/pyridoxamine kinase | 288 | 867 | 308 | null | - |
| **PAM435** | 483960 | 484565 | - | 39938486 | nfnB | nitroreductase | 201 | 606 | 291 | -0.113 | 0.952 |
| **PAM436** | 485212 | 485730 | + | 39938486 |  | hypothetical protein | 172 | 519 | 305 | -2.480 | 0.7488 |
| **PAM437** | 485736 | 485912 | + | 39938486 |  | hypothetical protein | 58 | 177 | 255 | insect | 0.4218 |
| **PAM438** | 486050 | 488065 | + | 39938486 | lig | NAD-dependent DNA ligase | 671 | 2016 | 280 | 0.887 | 0.996 |
| **PAM439** | 488140 | 489210 | + | 39938486 |  | uncharacterized BCR | 356 | 1071 | 307 | -1.477 | 0.4864 |
| **PAM440** | 489182 | 489565 | + | 39938486 | acpS | phosphopantetheinyl transferase | 127 | 384 | 286 | 0.013 | 0.9889 |
| **PAM441** | 489710 | 491053 | + | 39938486 | hcaD | uncharacterized NAD-dependent dehydrogenase | 447 | 1344 | 295 | 1.284 | 0.002949 |
| **PAM442** | 491244 | 491852 | + | 39938486 | sodA | superoxide dismutase | 202 | 609 | 282 | -0.550 | 0.4756 |
| **PAM443** | 491859 | 492308 | + | 39938486 | nusB | transcription termination factor | 149 | 450 | 296 | -2.101 | 0.03021 |
| **PAM444** | 492750 | 493565 | + | 39938486 |  | hypothetical protein | 271 | 816 | 288 | 1.150 | 0.002078 |
| **PAM445** | 494097 | 495914 | + | 39938486 | lepA | hypothetical protein | 605 | 1818 | 293 | -1.461 | 0.000611 |
| **PAM446** | 496013 | 496423 | - | 39938486 |  | hypothetical protein | 136 | 411 | 317 | null | - |
| **PAM447** | 496731 | 499487 | + | 39938486 | tldD | hypothetical protein | 918 | 2757 | 297 | null | 0.06597 |
| **PAM448** | 499642 | 502017 | - | 39938486 | lon | ATP-dependent Lon protease | 791 | 2376 | 291 | -0.412 | 0.713 |
| **PAM449** | 502104 | 503396 | - | 39938486 | tig | FKBP-type peptidyl-prolyl cis-trans isomerase | 430 | 1293 | 295 | 0.124 | 0.1627 |
| **PAM450** | 504067 | 506985 | - | 39938486 | uvrA | excinuclease ATPase subunit | 972 | 2919 | 313 | null | - |
| **PAM451** | 507953 | 508306 | + | 39938486 |  | hypothetical protein | 117 | 354 | 283 | -1.774 | 0.7481 |
| **PAM452** | 508313 | 508711 | + | 39938486 |  | hypothetical protein | 132 | 399 | 200 | 0.797 | 0.301 |
| **PAM453** | 509237 | 510310 | + | 39938486 | srmB | superfamily II DNA and RNA helicase | 357 | 1074 | 288 | null | - |
| **PAM454** | 510833 | 511711 | + | 39938486 | nfo | endonuclease IV | 292 | 879 | 319 | -0.968 | 0.1591 |
| **PAM455** | 512605 | 514278 | + | 39938486 | argS | arginyl-tRNA synthetase | 557 | 1674 | 282 | -1.330 | 0.000176 |
| **PAM457** | 516356 | 518185 | + | 39938486 | artI | ABC-type amino acid transport system, periplasmic component | 609 | 1830 | 284 | -1.321 | 0.02936 |
| **PAM458** | 520062 | 520499 | - | 39938486 |  | hypothetical protein | 145 | 438 | 280 | -1.626 | 0.009853 |
| **PAM459** | 520499 | 521515 | - | 39938486 |  | hypothetical protein | 338 | 1017 | 305 | 0.924 | 0.2633 |
| **PAM463** | 524180 | 524632 | + | 39938486 |  | hypothetical protein | 150 | 453 | 284 | 0.750 | - |
| **PAM465** | 525667 | 530382 | + | 39938486 | polC | DNA polymerase III alpha subunit | 1571 | 4716 | 291 | -0.459 | 0.01365 |
| **PAM466** | 530461 | 531498 | + | 39938486 | trpS | tryptophanyl-tRNA synthetase | 345 | 1038 | 302 | -0.139 | 0.6803 |
| **PAM467** | 531552 | 531995 | - | 39938486 | iscU | nifU homolog | 147 | 444 | 291 | 0.393 | 0.18 |
| **PAM468** | 532010 | 533215 | - | 39938486 | csdB | selenocysteine lyase | 401 | 1206 | 283 | -1.278 | 0.865 |
| **PAM469** | 533292 | 534533 | - | 39938486 | tyrS | tyrosyl-tRNA synthetase | 413 | 1242 | 293 | -1.784 | 0.3156 |
| **PAM470** | 534718 | 535074 | - | 39938486 | rplS | ribosomal protein L19 | 118 | 357 | 292 | 0.004 | 0.423 |
| **PAM471** | 535116 | 535844 | - | 39938486 | trmD | tRNA-(guanine-N1)-methyltransferase | 242 | 729 | 280 | -0.431 | 0.3847 |
| **PAM472** | 535904 | 536167 | - | 39938486 | rpsP | ribosomal protein S16 | 87 | 264 | 251 | -0.394 | 0.09962 |
| **PAM473** | 536618 | 537727 | - | 39938486 | prfB | protein chain release factor B | 369 | 1110 | 305 | 0.596 | 0.5069 |
| **PAM474** | 537731 | 540238 | - | 39938486 | secA | preprotein translocase subunit SecA | 835 | 2508 | 305 | 0.074 | 0.2131 |
| **PAM475** | 540618 | 540719 | + | 39938486 |  | hypothetical protein | 33 | 102 | 204 | -0.075 | 0.1166 |
| **PAM476** | 540857 | 541366 | - | 39938486 | mutT | MutT/nudix family protein | 169 | 510 | 284 | 0.034 | 0.9205 |
| **PAM477** | 542120 | 542692 | - | 39938486 |  | hypothetical protein | 190 | 573 | 288 | 1.153 | 0.4298 |
| **PAM478** | 544178 | 544810 | - | 39938486 |  | hypothetical protein | 210 | 633 | 306 | null | - |
| **PAM480** | 545871 | 546923 | - | 39938486 | hflB | ATP-dependent Zn protease | 350 | 1053 | 285 | null | - |
| **PAM481** | 548249 | 548740 | - | 39938486 |  | hypothetical protein | 163 | 492 | 318 | 1.134 | 0.7604 |
| **PAM482** | 548948 | 549406 | - | 39938486 |  | hypothetical protein | 152 | 459 | 291 | -0.561 | 0.925 |
| **PAM483** | 549445 | 550083 | - | 39938486 |  | hypothetical protein | 212 | 639 | 285 | -1.546 | 0.02549 |
| **PAM484** | 550810 | 551415 | + | 39938486 |  | hypothetical protein | 201 | 606 | 314 | -1.796 | 0.08254 |
| **PAM485** | 551560 | 552072 | - | 39938486 |  | hypothetical protein | 170 | 513 | 319 | -2.242 | 0.01777 |
| **PAM486** | 553541 | 554116 | + | 39938486 |  | hypothetical protein | 191 | 576 | 285 | -6.520 | 1.39E-07 |
| **PAM487** | 554510 | 555208 | + | 39938486 | artM | ABC-type amino acid transport system, permease component | 232 | 699 | 291 | -0.430 | 0.5185 |
| **PAM488** | 555201 | 555947 | + | 39938486 | glnQ | ABC-type polar amino acid transport system, ATPase component | 248 | 747 | 310 | -2.178 | 0.02205 |
| **PAM489** | 556151 | 557113 | + | 39938486 | artI | ABC-type amino acid transport system, periplasmic component | 320 | 963 | 281 | -1.056 | 0.7562 |
| **PAM490** | 557116 | 557850 | + | 39938486 | artM | ABC-type amino acid transport system, permease component | 244 | 735 | 281 | 0.766 | - |
| **PAM491** | 558137 | 558442 | + | 39938486 |  | hypothetical protein | 101 | 306 | 252 | null | - |
| **PAM493** | 565791 | 566372 | - | 39938486 |  | hypothetical protein | 193 | 582 | 310 | -1.666 | 0.5549 |
| **PAM494** | 566709 | 566987 | - | 39938486 | artM | ABC-type amino acid transport system, permease component | 92 | 279 | 261 | -0.320 | 0.4335 |
| **PAM495** | 567076 | 568083 | - | 39938486 | artI | ABC-type amino acid transport system, periplasmic component | 335 | 1008 | 311 | 0.700 | 0.7334 |
| **PAM496** | 568372 | 568701 | - | 39938486 |  | hypothetical protein | 109 | 330 | 308 | 2.335 | 0.3197 |
| **PAM498** | 569895 | 571910 | + | 39938486 | gyrB | DNA gyrase beta subunit | 671 | 2016 | 282 | 0.445 | 0.2877 |
| **PAM499** | 571995 | 574502 | + | 39938486 | gyrA | DNA gyrase alpha subunit | 835 | 2508 | 293 | 0.729 | 0.1072 |
| **PAM500** | 574597 | 575097 | + | 39938486 |  | hypothetical protein | 166 | 501 | 308 | -1.141 | 0.435 |
| **PAM501** | 575094 | 575807 | + | 39938486 |  | hypothetical protein | 237 | 714 | 284 | -0.196 | 0.705 |
| **PAM502** | 576161 | 577615 | + | 39938486 |  | hypothetical protein | 484 | 1455 | 288 | plant | 0.7164 |
| **PAM517** | 591617 | 592351 | + | 39938486 |  | hypothetical protein | 244 | 735 | 285 | null | - |
| **PAM518** | 592401 | 592808 | + | 39938486 |  | hypothetical protein | 135 | 408 | 299 | -5.583 | 0.1416 |
| **PAM522** | 595198 | 595809 | + | 39938486 |  | hypothetical protein | 203 | 612 | 282 | 4.825 | 6.53E-06 |
| **PAM539** | 612245 | 612976 | + | 39938486 |  | hypothetical protein | 243 | 732 | 291 | null | - |
| **PAM554** | 628185 | 628763 | - | 39938486 |  | hypothetical protein | 192 | 579 | 294 | null | - |
| **PAM555** | 629540 | 629872 | + | 39938486 |  | hypothetical protein | 110 | 333 | 287 | 2.054 | 0.05532 |
| **PAM558** | 631206 | 632285 | + | 39938486 |  | hypothetical protein | 359 | 1080 | 281 | 1.859 | 0.008222 |
| **PAM562** | 637270 | 637800 | - | 39938486 |  | hypothetical protein | 176 | 531 | 290 | -2.215 | 0.3254 |
| **PAM563** | 638113 | 638403 | - | 39938486 | himA | bacterial nucleoid DNA-binding protein | 96 | 291 | 250 | -0.683 | 0.09085 |
| **PAM564** | 638584 | 638919 | - | 39938486 | ssb | single-stranded DNA-binding protein | 111 | 336 | 295 | -1.519 | 0.2832 |
| **PAM565** | 639091 | 639975 | - | 39938486 | dam | site-specific DNA methylase | 294 | 885 | 301 | -1.226 | 0.09272 |
| **PAM573** | 649896 | 650522 | + | 39938486 |  | hypothetical protein | 208 | 627 | 289 | -0.350 | 0.8158 |
| **PAM575** | 652658 | 653392 | + | 39938486 |  | hypothetical protein | 244 | 735 | 296 | -1.562 | 0.00054 |
| **PAM577** | 654406 | 654894 | + | 39938486 |  | hypothetical protein | 162 | 489 | 286 | plant | - |
| **PAM579** | 656873 | 657574 | - | 39938486 | QRI7 | glycoprotein endopeptidase | 233 | 702 | 306 | -4.860 | 0.65 |
| **PAM580** | 657662 | 658177 | - | 39938486 | folK | 7,8-dihydro-6-hydroxymethylpterin- pyrophosphokinase | 171 | 516 | 306 | -3.226 | 0.2684 |
| **PAM581** | 658387 | 659268 | - | 39938486 | folP | dihydropteroate synthase | 293 | 882 | 287 | -1.136 | 0.9927 |
| **PAM582** | 659639 | 660238 | - | 39938486 |  | hypothetical protein | 199 | 600 | 293 | 0.591 | 0.07115 |
| **PAM583** | 660457 | 661323 | - | 39938486 | degV | uncharacterized BCR | 288 | 867 | 315 | -0.057 | 0.4734 |
| **PAM584** | 661452 | 662243 | - | 39938486 |  | uncharacterized ACR | 263 | 792 | 296 | -0.417 | 0.755 |
| **PAM585** | 662433 | 665111 | - | 39938486 | mgtA | cation transport ATPase | 892 | 2679 | 297 | -0.299 | 0.7148 |
| **PAM586** | 665484 | 665933 | - | 39938486 | rpsD | ribosomal protein S4 and related protein | 149 | 450 | 320 | -1.239 | 0.002013 |
| **PAM587** | 666680 | 667093 | + | 39938486 |  | hypothetical protein | 137 | 414 | 281 | -0.718 | 0.3662 |
| **PAM588** | 667259 | 668989 | - | 39938486 | artM | ABC-type amino acid transport system, permease component | 576 | 1731 | 280 | -1.311 | - |
| **PAM589** | 670622 | 672157 | + | 39938486 | lysU | lysyl-tRNA synthetase class II | 511 | 1536 | 282 | -0.484 | 0.2885 |
| **PAM590** | 672584 | 672946 | + | 39938486 |  | hypothetical protein | 120 | 363 | 318 | null | - |
| **PAM591** | 673050 | 674927 | + | 39938486 | dnaE | DNA polymerase III alpha subunit | 625 | 1878 | 289 | -0.774 | 0.4247 |
| **PAM592** | 676183 | 676779 | + | 39938486 |  | hypothetical protein | 198 | 597 | 319 | null | - |
| **PAM593** | 676893 | 677672 | + | 39938486 |  | hypothetical protein | 259 | 780 | 284 | -0.218 | 0.724 |
| **PAM594** | 677731 | 679407 | + | 39938486 | asnB | asparagine synthase | 558 | 1677 | 306 | -0.821 | 0.9505 |
| **PAM595** | 679611 | 679964 | + | 39938486 |  | hypothetical protein | 117 | 354 | 280 | -0.530 | 0.6886 |
| **PAM596** | 680030 | 681064 | + | 39938486 | pheS | phenylalanyl-tRNA synthetase alpha subunit | 344 | 1035 | 319 | null | - |
| **PAM597** | 681030 | 681575 | + | 39938486 |  | hypothetical protein | 181 | 546 | 296 | 1.108 | - |
| **PAM598** | 681568 | 683496 | + | 39938486 | pheT | phenylalanyl-tRNA synthetase beta subunit | 642 | 1929 | 284 | null | - |
| **PAM599** | 683685 | 685313 | + | 39938486 | phnL | ABC transporter, ATPase component | 542 | 1629 | 280 | null | - |
| **PAM600** | 685537 | 686628 | + | 39938486 | acoA | thiamine pyrophosphate-dependent dehydrogenase, E1 component alpha subunit | 363 | 1092 | 308 | 2.462 | 0.008435 |
| **PAM601** | 686631 | 687605 | + | 39938486 | acoB | thiamine pyrophosphate-dependent dehydrogenase, E1 component beta subunit | 324 | 975 | 288 | 2.341 | 0.07551 |
| **PAM602** | 687929 | 689113 | + | 39938486 | aceF | dihydrolipoamide acyltransferase | 394 | 1185 | 280 | 1.600 | 0.3385 |
| **PAM603** | 689135 | 690508 | + | 39938486 | lpd | fatty acid/phospholipid biosynthesis enzyme | 457 | 1374 | 281 | -0.014 | 0.2241 |
| **PAM604** | 690877 | 691233 | + | 39938486 |  | hypothetical protein | 118 | 357 | 250 | -0.825 | 0.3662 |
| **PAM605** | 691571 | 692338 | + | 39938486 | tatD | Mg-dependent DNase | 255 | 768 | 292 | 0.891 | 0.01854 |
| **PAM606** | 692433 | 692768 | - | 39938486 |  | hypothetical protein | 111 | 336 | 289 | -1.173 | 0.1543 |
| **PAM607** | 693444 | 694544 | + | 39938486 | plsX | fatty acid/phospholipid biosynthesis enzyme | 366 | 1101 | 286 | -1.469 | 0.7638 |
| **PAM608** | 694541 | 695257 | + | 39938486 | rnc | dsRNA-specific ribonuclease | 238 | 717 | 282 | -0.927 | 0.775 |
| **PAM609** | 695199 | 695882 | + | 39938486 |  | hypothetical protein | 227 | 684 | 318 | 0.773 | 0.1784 |
| **PAM610** | 696009 | 696476 | + | 39938486 |  | hypothetical protein | 155 | 468 | 285 | 0.157 | 0.1669 |
| **PAM611** | 696557 | 698179 | - | 39938486 | pyrG | CTP synthase | 540 | 1623 | 280 | 0.488 | 0.9585 |
| **PAM612** | 698172 | 699170 | - | 39938486 | psd | phosphatidylserine decarboxylase | 332 | 999 | 306 | -0.204 | 0.6489 |
| **PAM613** | 699183 | 699917 | - | 39938486 | pssA | phosphatidylserine synthase | 244 | 735 | 284 | -1.267 | 0.912 |
| **PAM614** | 700093 | 700602 | - | 39938486 |  | hypothetical protein | 169 | 510 | 282 | -0.115 | 0.5334 |
| **PAM615** | 700803 | 701012 | - | 39938486 |  | uncharacterized BCR | 69 | 210 | 250 | null | - |
| **PAM616** | 701025 | 701519 | - | 39938486 |  | hypothetical protein | 164 | 495 | 304 | -0.698 | 0.1082 |
| **PAM617** | 701516 | 702964 | - | 39938486 | dnaX | DNA polymerase III, gamma/tau subunit | 482 | 1449 | 286 | -0.806 | 0.832 |
| **PAM618** | 704872 | 705471 | + | 39938486 | hsdR | restriction enzymes type I helicase subunit | 199 | 600 | 292 | -0.343 | - |
| **PAM619** | 707482 | 707850 | + | 39938486 | hsdM | type I restriction-modification system methyltransferase subunit | 122 | 369 | 291 | null | 0.08621 |
| **PAM620** | 709398 | 711212 | + | 39938486 | glnS | glutamyl- and glutaminyl-tRNA synthetase | 604 | 1815 | 287 | null | - |
| **PAM621** | 711332 | 713575 | + | 39938486 | uvrD | ATP-dependent DNA helicase | 747 | 2244 | 287 | -0.746 | - |
| **PAM622** | 713917 | 714495 | + | 39938486 |  | hypothetical protein | 192 | 579 | 307 | null | - |
| **PAM623** | 714724 | 714897 | + | 39938486 | rpsU | ribosomal protein S21 | 57 | 174 | 200 | -1.736 | 0.000336 |
| **PAM624** | 715119 | 715559 | + | 39938486 |  | hypothetical protein | 146 | 441 | 297 | -1.280 | 0.1458 |
| **PAM625** | 715683 | 716570 | + | 39938486 | era | glycyl-tRNA synthetase | 295 | 888 | 288 | null | - |
| **PAM626** | 716670 | 718028 | + | 39938486 | GRS1 | glycyl-tRNA synthetase, class II | 452 | 1359 | 280 | -0.635 | 0.7509 |
| **PAM627** | 718038 | 719852 | + | 39938486 | dnaG | DNA primase | 604 | 1815 | 281 | -0.174 | 0.5386 |
| **PAM628** | 719935 | 721317 | + | 39938486 | rpoD | DNA-directed RNA polymerase sigma subunit | 460 | 1383 | 280 | 1.041 | 0.04457 |
| **PAM629** | 721319 | 721762 | + | 39938486 |  | hypothetical protein | 147 | 444 | 293 | -2.315 | 0.1122 |
| **PAM630** | 722036 | 722497 | - | 39938486 | hit | diadenosine tetraphosphate hydrolase | 153 | 462 | 288 | null | - |
| **PAM631** | 722519 | 723511 | - | 39938486 |  | ribonuclease HII Family 2 | 330 | 993 | 288 | null | - |
| **PAM632** | 724063 | 724368 | - | 39938486 |  | hypothetical protein | 101 | 306 | 282 | 0.244 | 0.636 |
| **PAM633** | 725007 | 726698 | - | 39938486 |  | hypothetical protein | 563 | 1692 | 291 | -0.141 | 0.0688 |
| **PAM634** | 726891 | 727697 | + | 39938486 |  | hypothetical protein | 268 | 807 | 295 | -2.722 | 0.1545 |
| **PAM636** | 728529 | 729392 | - | 39938486 | degV | uncharacterized BCR | 287 | 864 | 282 | -0.658 | 0.03422 |
| **PAM637** | 729728 | 731452 | - | 39938486 |  | hypothetical protein | 574 | 1725 | 308 | 0.831 | 0.5149 |
| **PAM638** | 731545 | 731664 | + | 39938486 |  | hypothetical protein | 39 | 120 | 250 | -0.385 | 0.8054 |
| **PAM639** | 732710 | 733468 | - | 39938486 | artM | ABC-type amino acid transport system, permease component | 252 | 759 | 294 | null | - |
| **PAM640** | 733781 | 734584 | - | 39938486 |  | hypothetical protein | 267 | 804 | 304 | null | - |
| **PAM641** | 734556 | 734930 | - | 39938486 | mdoB | amino acid transporter, amino acid-binding protein | 124 | 375 | 280 | null | - |
| **PAM642** | 735313 | 736251 | - | 39938486 | tra5 | putative transposase | 312 | 939 | 292 | -0.846 | 0.03113 |
| **PAM649** | 743119 | 743394 | - | 39938486 |  | hypothetical protein | 91 | 276 | 219 | -0.509 | 0.3275 |
| **PAM652** | 745341 | 746201 | - | 39938486 |  | hypothetical protein | 286 | 861 | 304 | null | - |
| **PAM657** | 750626 | 750940 | - | 39938486 |  | hypothetical protein | 104 | 315 | 282 | -2.649 | - |
| **PAM659** | 754427 | 755680 | + | 39938486 | potA | ABC-type spermidine/putrescine transport systems, ATPase component | 417 | 1254 | 284 | null | - |
| **PAM660** | 755674 | 756522 | + | 39938486 | potB | ABC-type spermidine/putrescine transport system, permease component I | 282 | 849 | 295 | null | - |
| **PAM661** | 756512 | 757516 | + | 39938486 | potC | ABC-type spermidine/putrescine transport system, permease component II | 334 | 1005 | 315 | 1.691 | 0.173 |
| **PAM662** | 757513 | 758739 | + | 39938486 | potD | spermidine/putrescine-binding periplasmic protein | 408 | 1227 | 303 | null | - |
| **PAM663** | 758935 | 759948 | - | 39938486 |  | hypothetical protein | 337 | 1014 | 284 | 1.176 | - |
| **PAM664** | 760408 | 760851 | - | 39938486 | rpt1 | ATP-dependent 26S proteasome regulatory subunit | 147 | 444 | 320 | null | - |
| **PAM665** | 761746 | 763461 | + | 39938486 |  | hypothetical protein | 571 | 1716 | 298 | -0.688 | 0.4159 |
| **PAM668** | 766890 | 767249 | + | 39938486 |  | hypothetical protein | 119 | 360 | 287 | -0.270 | 0.6474 |
| **PAM669** | 767348 | 767890 | - | 39938486 |  | hypothetical protein | 180 | 543 | 281 | -1.620 | 0.1545 |
| **PAM670** | 767954 | 768355 | - | 39938486 |  | hypothetical protein | 133 | 402 | 200 | -0.607 | 0.1101 |
| **PAM671** | 769504 | 769728 | + | 39938486 | cspC | cold shock protein | 74 | 225 | 212 | -1.375 | 0.000254 |
| **PAM672** | 770513 | 771331 | - | 39938486 | dnaC | DNA replication protein | 272 | 819 | 296 | -1.787 | 0.06844 |
| **PAM673** | 771414 | 772607 | - | 39938486 | dnaB | replication initiation/membrane attachment protein | 397 | 1194 | 304 | -0.610 | 0.0907 |
| **PAM674** | 772661 | 773491 | - | 39938486 | nei | formamidopyrimidine-DNA glycosylase | 276 | 831 | 293 | -0.734 | 0.3769 |
| **PAM675** | 773647 | 775089 | - | 39938486 | polA | DNA polymerase I | 480 | 1443 | 304 | -6.977 | 0.07055 |
| **PAM676** | 775265 | 776233 | - | 39938486 | exo | 5'-3' exonuclease | 322 | 969 | 294 | -0.545 | 0.8905 |
| **PAM677** | 776341 | 777396 | - | 39938486 |  | hypothetical protein | 351 | 1056 | 288 | -0.972 | 0.02793 |
| **PAM678** | 777633 | 778913 | - | 39938486 | ffh | signal recognition particle GTPase | 426 | 1281 | 285 | -1.549 | 0.03641 |
| **PAM679** | 779175 | 780218 | - | 39938486 | ftsY | signal recognition particle-docking protein FtsY | 347 | 1044 | 285 | -4.554 | 0.2735 |
| **PAM680** | 780557 | 781078 | - | 39938486 | gmk | guanylate kinase | 173 | 522 | 280 | -1.845 | 0.02734 |
| **PAM683** | 783244 | 784191 | + | 39938486 | tra5 | putative transposase | 315 | 948 | 305 | null | - |
| **PAM685** | 785385 | 786710 | - | 39938486 | dnaG | DNA primase | 441 | 1326 | 284 | plant | - |
| **PAM686** | 786737 | 788236 | - | 39938486 | dnaB | replicative DNA helicase | 499 | 1500 | 282 | null | - |
| **PAM687** | 788254 | 788865 | - | 39938486 |  | hypothetical protein | 203 | 612 | 299 | 0.072 | 0.5406 |
| **PAM688** | 789030 | 789659 | - | 39938486 | tmk | thymidylate kinase | 209 | 630 | 283 | -1.595 | 0.132 |
| **PAM690** | 790457 | 790720 | - | 39938486 |  | hypothetical protein | 87 | 264 | 243 | -1.871 | 0.004772 |
| **PAM692** | 791628 | 793319 | - | 39938486 |  | hypothetical protein | 563 | 1692 | 280 | null | - |
| **PAM693** | 793291 | 794121 | - | 39938486 | smc | chromosome segregation ATPase homolog | 276 | 831 | 280 | null | - |
| **PAM694** | 794054 | 794995 | - | 39938486 |  | hypothetical protein | 313 | 942 | 289 | null | - |
| **PAM695** | 795016 | 796524 | - | 39938486 | hflB | ATP-dependent Zn protease | 502 | 1509 | 318 | -2.931 | 0.2768 |
| **PAM697** | 797512 | 797955 | - | 39938486 |  | hypothetical protein | 147 | 444 | 250 | -1.248 | 0.9852 |
| **PAM698** | 798267 | 798599 | - | 39938486 | himA | bacterial nucleoid DNA-binding protein | 110 | 333 | 281 | null | - |
| **PAM699** | 798672 | 798986 | - | 39938486 | ssb | single-stranded DNA-binding protein | 104 | 315 | 302 | -0.709 | 0.6538 |
| **PAM700** | 799001 | 799687 | - | 39938486 | fliA | DNA-directed RNA polymerase specialized sigma subunit | 228 | 687 | 317 | plant | - |
| **PAM701** | 799898 | 800134 | - | 39938486 |  | hypothetical protein | 78 | 237 | 200 | -1.407 | - |
| **PAM702** | 800136 | 800762 | - | 39938486 | rpoD | DNA-directed RNA polymerase sigma subunit | 208 | 627 | 280 | null | - |
| **PAM703** | 801973 | 802746 | - | 39938486 | dnaJ | molecular chaperone DnaJ | 257 | 774 | 292 | -0.710 | 0.1543 |
| **PAM704** | 802898 | 804763 | - | 39938486 | dnaK | molecular chaperone DnaK | 621 | 1866 | 289 | 1.806 | 0.03086 |
| **PAM705** | 804729 | 805472 | - | 39938486 | grpE | molecular chaperone GrpE | 247 | 744 | 280 | 0.207 | 0.3885 |
| **PAM706** | 805694 | 806722 | - | 39938486 | hrcA | heat-inducible transcription repressor | 342 | 1029 | 294 | -0.430 | 0.08242 |
| **PAM707** | 808486 | 809133 | + | 39938486 |  | hypothetical protein | 215 | 648 | 287 | 1.881 | 0.1131 |
| **PAM708** | 809411 | 809623 | + | 39938486 |  | hypothetical protein | 70 | 213 | 202 | -0.514 | 0.9472 |
| **PAM709** | 810263 | 810463 | + | 39938486 |  | hypothetical protein | 66 | 201 | 234 | 0.054 | 0.3521 |
| **PAM710** | 810537 | 810977 | + | 39938486 |  | hypothetical protein | 146 | 441 | 280 | -0.197 | 0.2516 |
| **PAM711** | 811244 | 811837 | + | 39938486 |  | hypothetical protein | 197 | 594 | 251 | -0.002 | 0.228 |
| **PAM712** | 811921 | 812499 | + | 39938486 |  | hypothetical protein | 192 | 579 | 293 | null | - |
| **PAM714** | 814483 | 816141 | + | 39938486 | srmB | superfamily II DNA and RNA helicase | 552 | 1659 | 286 | -0.142 | 0.5071 |
| **PAM715** | 816273 | 816458 | - | 39938486 |  | hypothetical protein | 61 | 186 | 281 | -0.488 | 0.07794 |
| **PAM716** | 816582 | 816848 | - | 39938486 |  | hypothetical protein | 88 | 267 | 250 | 0.181 | 0.156 |
| **PAM717** | 816915 | 817268 | - | 39938486 |  | hypothetical protein | 117 | 354 | 280 | null | - |
| **PAM718** | 817523 | 818182 | - | 39938486 |  | phosphatidylglycerophosphate synthase | 219 | 660 | 310 | -4.361 | - |
| **PAM719** | 818163 | 818318 | - | 39938486 |  | hypothetical protein | 51 | 156 | 228 | null | - |
| **PAM720** | 818800 | 819915 | - | 39938486 | citS | malate/citrate symporter | 371 | 1116 | 301 | 0.455 | 0.02451 |
| **PAM721** | 820334 | 821506 | - | 39938486 | sfcA | malic enzyme | 390 | 1173 | 309 | 0.362 | 0.7488 |
| **PAM723** | 822461 | 823672 | - | 39938486 |  | hypothetical protein | 403 | 1212 | 316 | 1.036 | 0.01351 |
| **PAM724** | 823693 | 823911 | - | 39938486 |  | hypothetical protein | 72 | 219 | 201 | null | 0.6904 |
| **PAM726** | 825777 | 826106 | + | 39938486 | ssb | single-stranded DNA-binding protein | 109 | 330 | 303 | 0.276 | 0.7703 |
| **PAM729** | 830614 | 831210 | + | 39938486 |  | hypothetical protein | 198 | 597 | 292 | 0.401 | 0.785 |
| **PAM730** | 831517 | 832770 | + | 39938486 |  | HD superfamily phosphohydrolase | 417 | 1254 | 317 | 1.276 | 0.1216 |
| **PAM731** | 832877 | 833683 | + | 39938486 | ksgA | dimethyladenosine transferase | 268 | 807 | 286 | -0.547 | 0.7996 |
| **PAM732** | 833959 | 834258 | + | 39938486 |  | hypothetical protein | 99 | 300 | 280 | 0.358 | 0.534 |
| **PAM733** | 834478 | 836478 | + | 39938486 | zntA | cation transport ATPase | 666 | 2001 | 291 | -2.821 | 0.2965 |
| **PAM734** | 836678 | 838177 | - | 39938486 | norM | Na+-driven multidrug efflux pump | 499 | 1500 | 280 | 0.140 | 0.03511 |
| **PAM735** | 838300 | 839328 | - | 39938486 |  | hypothetical protein | 342 | 1029 | 291 | null | - |
| **PAM736** | 839433 | 839630 | - | 39938486 | rpoZ | DNA-directed RNA polymerase subunit K/omega | 65 | 198 | 293 | -0.337 | 0.7732 |
| **PAM737** | 839611 | 840249 | - | 39938486 | gmk | guanylate kinase | 212 | 639 | 280 | 0.719 | 0.01529 |
| **PAM738** | 840400 | 842865 | - | 39938486 | leuS | leucyl-tRNA synthetase | 821 | 2466 | 303 | 0.304 | 0.2308 |
| **PAM739** | 842969 | 843199 | - | 39938486 | acpP | acyl carrier protein | 76 | 231 | 202 | null | 0.04233 |
| **PAM740** | 843470 | 844534 | - | 39938486 | frvX | endo-1,4-beta-glucanase homolog | 354 | 1065 | 290 | -0.950 | 0.1289 |
| **PAM741** | 844521 | 845150 | - | 39938486 |  | hypothetical protein | 209 | 630 | 301 | -1.157 | 0.763 |
| **PAM742** | 845289 | 845912 | - | 39938486 | rplT | ribosomal protein L20 | 207 | 624 | 293 | 0.678 | 0.2912 |
| **PAM743** | 845927 | 846133 | - | 39938486 | rpmI | ribosomal protein L35 | 68 | 207 | 200 | 0.197 | 0.3119 |
| **PAM744** | 846266 | 846718 | - | 39938486 | infC | translation initiation factor IF3 | 150 | 453 | 281 | null | - |
| **PAM745** | 847449 | 848375 | - | 39938486 | thrS | threonyl-tRNA synthetase | 308 | 927 | 310 | 0.276 | 0.1751 |
| **PAM746** | 849379 | 850317 | - | 39938486 |  | hypothetical protein | 312 | 939 | 300 | 0.998 | 0.01907 |
| **PAM747** | 850416 | 851798 | - | 39938486 | thdF | tRNA modification GTPase | 460 | 1383 | 284 | 0.720 | 0.5593 |
| **PAM748** | 852017 | 852985 | - | 39938486 |  | exopolyphosphatase-related protein | 322 | 969 | 295 | -1.040 | 0.1083 |
| **PAM749** | 853020 | 854642 | - | 39938486 | ugpB | sugar-binding periplasmic protein | 540 | 1623 | 287 | 0.265 | 0.1862 |
| **PAM750** | 854626 | 855420 | - | 39938486 | ugpE | sugar permease | 264 | 795 | 283 | null | - |
| **PAM751** | 855549 | 856490 | - | 39938486 | ugpA | ABC-type sugar transport system, permease component | 313 | 942 | 306 | 1.069 | 0.1722 |
| **PAM752** | 856554 | 857597 | - | 39938486 | malK | ABC-type sugar transport system, ATPase component | 347 | 1044 | 280 | -0.778 | 0.5153 |
| **PAM753** | 857848 | 857964 | - | 39938486 |  | hypothetical protein | 38 | 117 | 210 | 0.461 | 0.5674 |
| **PAM754** | 858081 | 859601 | - | 39938486 | asnS | aspartyl/asparaginyl-tRNA synthetase | 506 | 1521 | 280 | 1.191 | 0.758 |
