# Supplementary material for: Dramatic Transcriptional Changes in an Intracellular Parasite Enable Host Switching between Plant and Insect
Source: PLoS One. 2011 Aug 16;6(8):e23242. doi: 10.1371/journal.pone.0023242 (PMC3156718; doi:10.1371/journal.pone.0023242)
Supplement: Table S2 — Primer sequence list used for qRT-PCR. (DOC) [file pone.0023242.s006.doc]

Table S2

Primer sequence list used for qRT-PCR.

| **Primer name** | **Direction** | **Sequence** | **Purpose** |
| --- | --- | --- | --- |
| **PAM028_F** | forward | AGAAGATGTTAAAGTTGCTTTGG | qRT-PCR for PAM028 |
| **PAM028_R** | reverse | AAATCCTTGAAATCTTTCTTTACG | qRT-PCR for PAM028 |
| **PAM058_F** | forward | TAGCTGTTGCAGTTGTAATAGGACA | qRT-PCR for PAM058 |
| **PAM058_R** | reverse | AAACAACAAAGAAAAAAGAGGCA | qRT-PCR for PAM058 |
| **PAM092_F** | forward | GGTGATGTGGCAGAAGAAGAAC | qRT-PCR for PAM092 |
| **PAM092_R** | reverse | GTGCGGATCAATTCCTACTCC | qRT-PCR for PAM092 |
| **PAM096_F** | forward | AGGGGTTTCTGATCGAGATATTCAC | qRT-PCR for PAM096 |
| **PAM096_R** | reverse | GCCTTGCAAAGAAGCTGATATTACC | qRT-PCR for PAM096 |
| **PAM122_F** | forward | TGTTGCTTTAATGTTTGTTGGCG | qRT-PCR for PAM122 |
| **PAM122_R** | reverse | AGCAGCAGTAAGTTCAAGAGCGTC | qRT-PCR for PAM122 |
| **PAM242_F** | forward | TTTGGCACCTTTGAAGTAAAACAC | qRT-PCR for PAM242 |
| **PAM242_R** | reverse | TGAAAGTAGGAACCTTTTTAGCTGG | qRT-PCR for PAM242 |
| **PAM266_F** | forward | GTGTTGTCTTTATGCAGCATTTGG | qRT-PCR for PAM266 |
| **PAM266_R** | reverse | TTCGGTGTAATCAAAATGAAGTGC | qRT-PCR for PAM266 |
| **PAM271_R** | forward | TAGAAGAAGAATCTCCTCAAGGGTC | qRT-PCR for PAM271 |
| **PAM271_R** | reverse | GCTGGGTTTGGGTTTTGTG | qRT-PCR for PAM271 |
| **PAM289_R** | forward | TTTGAAAGTGCACCAAATGAC | qRT-PCR for PAM289 |
| **PAM289_R** | reverse | AAATCGTCTCCAATAGAAAGACC | qRT-PCR for PAM289 |
| **PAM442_F** | forward | TGATTGACTGCGAATTTGGTTC | qRT-PCR for PAM442 |
| **PAM442_R** | reverse | AGTTAATACAAGCCAAGCCCAAC | qRT-PCR for PAM442 |
| **PAM485_F** | forward | GTGCAAGAAGCTTTAGACCAATTG | qRT-PCR for PAM485 |
| **PAM485_R** | reverse | CACCTGAAGGAGTTAAAGGATTTTG | qRT-PCR for PAM485 |
| **PAM486_R** | forward | CACAATCACAAGGACCAAAATTAGAG | qRT-PCR for PAM486 |
| **PAM486_R** | reverse | GTAAGCCACGCAAGAACATGATAA | qRT-PCR for PAM486 |
| **PAM600_F** | forward | TTATGCTTGTGGAATTCCTGGC | qRT-PCR for PAM600 |
| **PAM600_R** | reverse | TTCGTTAAAGGCTTCTTGGGC | qRT-PCR for PAM600 |
| **PAM606_F** | forward | GCAACCAGGACAAGTTATTAAAGTG | qRT-PCR for PAM606 |
| **PAM606_R** | reverse | CAGTTTGGCGAAAACTATCTACATC | qRT-PCR for PAM606 |
| **PAM726_R** | forward | CGTCTCAGCCATGATTTAGAAAAAC | qRT-PCR for PAM726 |
| **PAM726_R** | reverse | CACATGGAATGAATTGGGTTATCTC | qRT-PCR for PAM726 |
| **PAM733_F** | forward | TCTATATTTGCATTCTCACAATTGC | qRT-PCR for PAM733 |
| **PAM733_R** | reverse | GATAGCCGATTAAAAATAAAAGTGC | qRT-PCR for PAM733 |
| **PAM745_F** | forward | GAGCTACTGTAAGGCGCATCG | qRT-PCR for PAM745 |
| **PAM745_R** | reverse | TGGGCGTATAGACATGATGATAATC | qRT-PCR for PAM745 |
